# Activation of GPR81 by lactate drives tumour-induced cachexia

**DOI:** 10.1038/s42255-024-01011-0

**Published:** 2024-03-18

**Authors:** Xidan Liu, Shijin Li, Qionghua Cui, Bujing Guo, Wanqiu Ding, Jie Liu, Li Quan, Xiaochuan Li, Peng Xie, Li Jin, Ye Sheng, Wenxin Chen, Kai Wang, Fanxin Zeng, Yifu Qiu, Changlu Liu, Yan Zhang, Fengxiang Lv, Xinli Hu, Rui-Ping Xiao

**Affiliations:** 1 Institute of Molecular Medicine, College of Future Technology, Peking University, Beijing, China; 2 Dazhou Central Hospital, Sichuan, China; 3 Peking-Tsinghua Center for Life Sciences, Peking University, Beijing, China; 4 Sanford Burnham Prebys Medical Discovery Institute, La Jolla, CA USA; 5 State Key Laboratory of Membrane Biology, Peking University, Beijing, China; 6 Beijing City Key Laboratory of Cardiometabolic Molecular Medicine, Peking University, Beijing, China; 7 PKU-Nanjing Institute of Translational Medicine, Nanjing, China

**Keywords:** Cancer, Molecular medicine, Homeostasis, Cancer metabolism

## Abstract

Cachexia affects 50–80% of patients with cancer and accounts for 20% of cancer-related death, but the underlying mechanism driving cachexia remains elusive. Here we show that circulating lactate levels positively correlate with the degree of body weight loss in male and female patients suffering from cancer cachexia, as well as in clinically relevant mouse models. Lactate infusion per se is sufficient to trigger a cachectic phenotype in tumour-free mice in a dose-dependent manner. Furthermore, we demonstrate that adipose-specific G-protein-coupled receptor (GPR)81 ablation, similarly to global GPR81 deficiency, ameliorates lactate-induced or tumour-induced adipose and muscle wasting in male mice, revealing adipose GPR81 as the major mediator of the catabolic effects of lactate. Mechanistically, lactate/GPR81-induced cachexia occurs independently of the well-established protein kinase A catabolic pathway, but it is mediated by a signalling cascade sequentially activating Gi–Gβγ–RhoA/ROCK1–p38. These findings highlight the therapeutic potential of targeting GPR81 for the treatment of this life-threatening complication of cancer.

## Abstract (web summary)

Tumour-derived lactate activates adipose GPR81, which in turn leads to cachexia. Targeting GPR81 and its downstream signalling pathway holds therapeutic potential for treating cancer cachexia.

## Main

Cachexia is featured by a rapid reduction of body weight and accounts for about 20% of cancer-related death^1^. Patients with cancer cachexia experience asthenia, anorexia, anaemia and fatigue, resulting in deterioration of quality of life and poor tolerance to cancer therapies^2^. While cancer cachexia exemplifies an outstanding unmet medical need, its underlying mechanism is poorly understood^3^.

Losses of fat and muscle mass are the key manifestations of cachexia. Several inflammatory cytokines, such as tumour necrosis factor (TNF), interleukin (IL)-6, transforming growth factor-β and interferon (IFN)-γ, have been implicated in stimulating adipose and muscle remodelling caused by exuberant growth of cancer cells. They are considered as major drivers for the pathogenesis of cancer cachexia. However, the results of multiple clinical trials of anti-inflammation treatments are disappointing, suggesting that targeting inflammatory cytokines is not adequate to cure cancer cachexia^4^. To date, there is still a fundamental knowledge gap in the field of cancer biology and medicine, namely the link between tumour and maladaptation of host metabolism. The present study seeks to identify the causal factor connecting tumour with the metabolic and structural remodelling of adipose tissue and skeletal muscle.

Using unbiased screening of metabolites in the blood from patients and animal models with cancer cachexia, in conjunction with genetic and pharmacological approaches, we have pinpointed lactate as a causal factor of cancer cachexia. Furthermore, we have demonstrated that G-protein-coupled receptor (GPR)81, but not other lactate sensors, as a key mediator of lactate signalling; which in turn, is transduced by the Gαi/o-Gβγ–RhoA/ROCK1–p38 cascade to activate thermogenic and lipolytic programmes of adipose tissue, independent of the classic adenylyl cyclase–cAMP–protein kinase A (PKA) pathway. These findings have defined lactate as a necessary and sufficient link between tumour and adipose catabolism and subsequent muscle wasting, revealing lactate–GPR81 signalling as a potential therapeutic target for the vicious cancer complication, cancer cachexia.

## Circulating lactate level increases in cancer cachexia

**Fig. 1:**
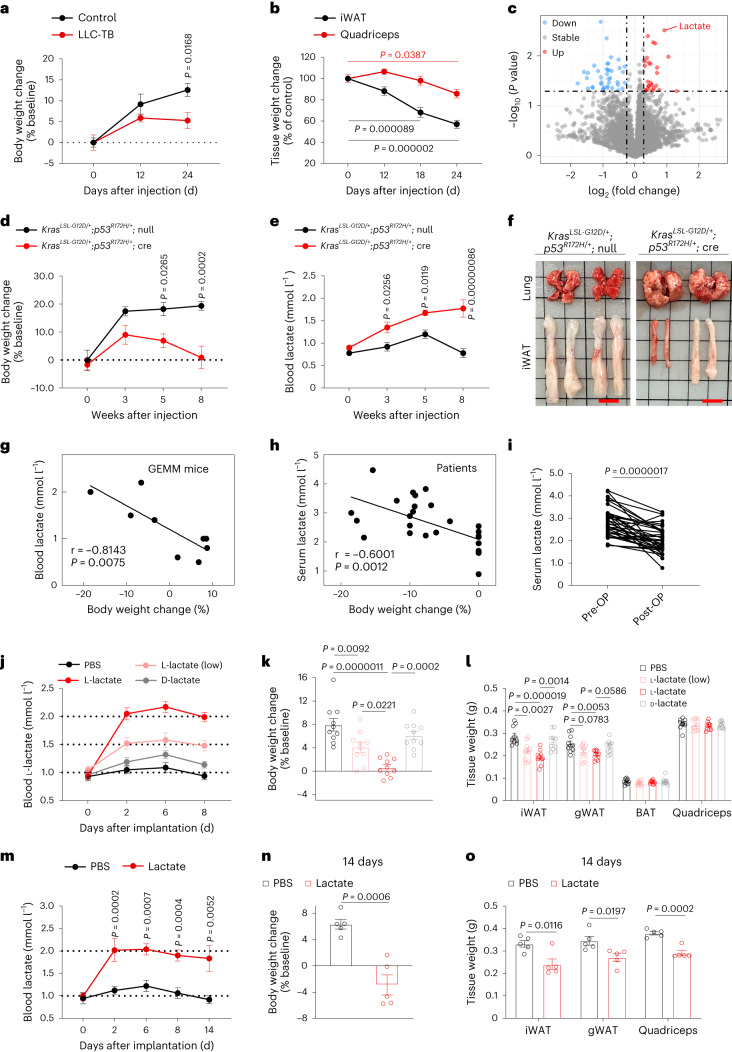
Lactate induces cachectic phenotype. **a**, Body weights of tumour-free (control) and LLC tumour-bearing (LLC-TB) mice after xenoplantation with LLC cells subcutaneously (*n* = 5 for each group). **b**, Tissue weights of iWAT and quadriceps of mice injected with LLC cells or PBS (*n* = 5 for each group). **c**, Volcano plot showing differential metabolites in the serum that were significantly increased (red) or decreased (blue) in the LLC-TB mice compared to control mice (*n* = 4 for each group). **d**, Body weights of *Kras*^*LSL-G12D/+*^;*p53*^*R172H/+*^ mice after intranasal administration of control adenovirus (*Kras*^*LSL-G12D/+*^;*p53*^*R172H/+*^;null; *n* = 5) or adenovirus expressing Cre recombinase (*Kras*^*LSL-G12D/+*^;*p53*^*R172H/+*^;Cre; *n* = 4). **e**, Blood lactate levels of *Kras*^*LSL-G12D/+*^;*p53*^*R172H/+*^;null (*n* = 5) or *Kras*^*LSL-G12D/+*^;*p53*^*R172H/+*^;Cre mice (*n* = 4). **f**, Representative anatomical images of lung and iWAT from *Kras*^*LSL-G12D/+*^;*p53*^*R172H/+*^;null and *Kras*^*LSL-G12D/+*^;*p53*^*R172H/+*^;Cre mice. Scale bars, 1 cm. **g**, Linear regression analysis showing the correlation between blood lactate level and percentage of body weight change in the *Kras*^*LSL-G12D/+*^;*p53*^*R172H/+*^ mice (*n* = 9). **h**, Linear regression analysis showing the correlation between serum lactate level and percentage of body weight change in patients with lung adenocarcinoma (*n* = 26). **i**, The serum lactate level in patients with lung adenocarcinoma before surgical removal of tumour (pre-OP) and 1 month after the operation (post-OP; *n* = 36). **j**–**l**, Blood lactate levels (**j**), body weight change (**k**) and weights of iWAT, gWAT, BAT and quadriceps (**l**) in the mice implanted with osmotic minipumps (*n* = 10 for each group). **m**–**o**, Blood lactate levels (**m**; day 2, 6, 8, *n* = 10; day 14, *n* = 6), body weight change (**n**; *n* = 5 for each group) and tissue weights (**o**; *n* = 5 for each group) in the mice implanted with osmotic minipumps. All data are presented as the mean ± s.e.m. *P* values were determined by two-way analysis of variance (ANOVA; **a**, **d**, **e**, **j** and **m**), one-way ANOVA with Tukey’s multiple-comparisons test (**b**, **k** and **l**) or two-tailed unpaired Student’s *t*-test (**i**, **n** and **o**). *P* values of the correlations were determined by two-tailed Pearson correlation analysis (**g** and **h**). Source data

**Extended Data Fig. 1:**
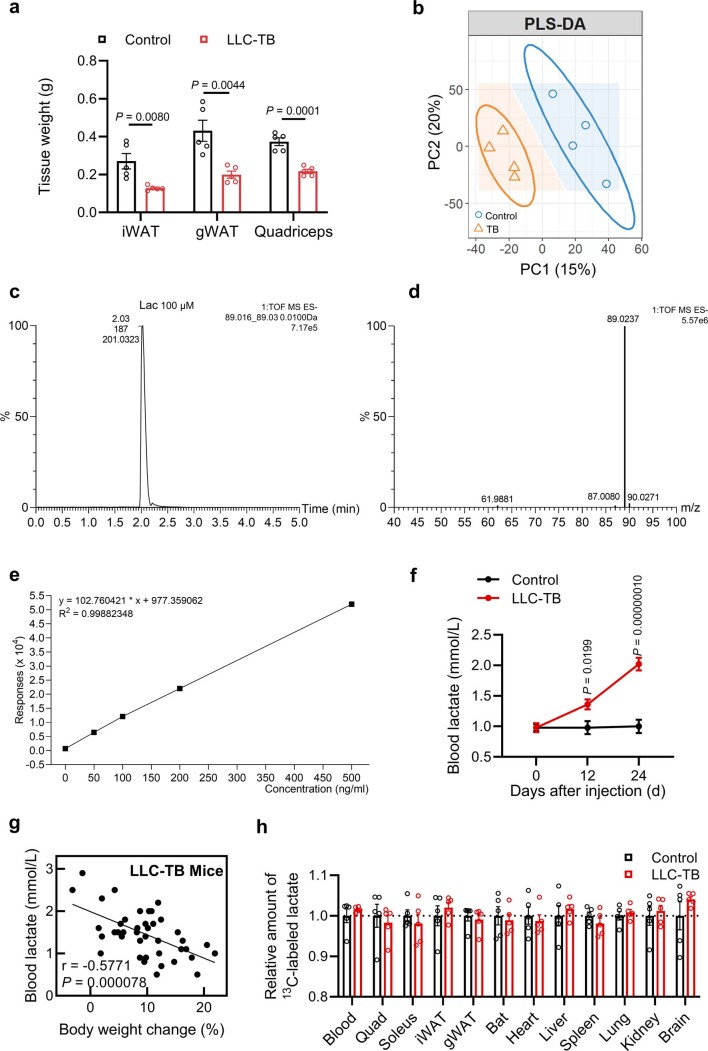
Differential serum metabolites in tumour-free versus tumour-bearing mice. **a**, Tissue weights of inguinal white adipose tissue (iWAT), gonad white adipose tissue (gWAT), and quadriceps of tumour-free (Control) and xenograft tumour-bearing mice (LLC-TB) 24 days after injected with LLC cells (n = 5 for each group). **b**, Partial Least Squares Discriminant Analysis (PLS-DA) of untargeted metabolomics profiling of serum samples from the LLC-TB and Control mice (n = 4 for each group). **c-e**, The retention time (**c**), m/z value (**d**), and calibration curve (**e**) of lactate standard in mass spectrum. **f**, Blood lactate levels of Control and LLC-TB mice on day 0, 12 and 24 days after injecting PBS or LLC cells (n = 5 for each group). **g**, Linear regression analysis showing the correlation between blood lactate level and percentage of body weight change in the mice xenograft with LLC cells (n = 41). **h**, Relative amount of ^13^C-labeled lactate in the blood and major organs of mice with or without tumour (n = 5). All data are presented as the mean ± s.e.m. *P* values were determined by two-tailed unpaired Student’s *t*-test (a, h), two-way ANOVA (f). *P* value of the correlation was determined by two-tailed Pearson correlation analysis (g). Source data

**Extended Data Fig. 2:**
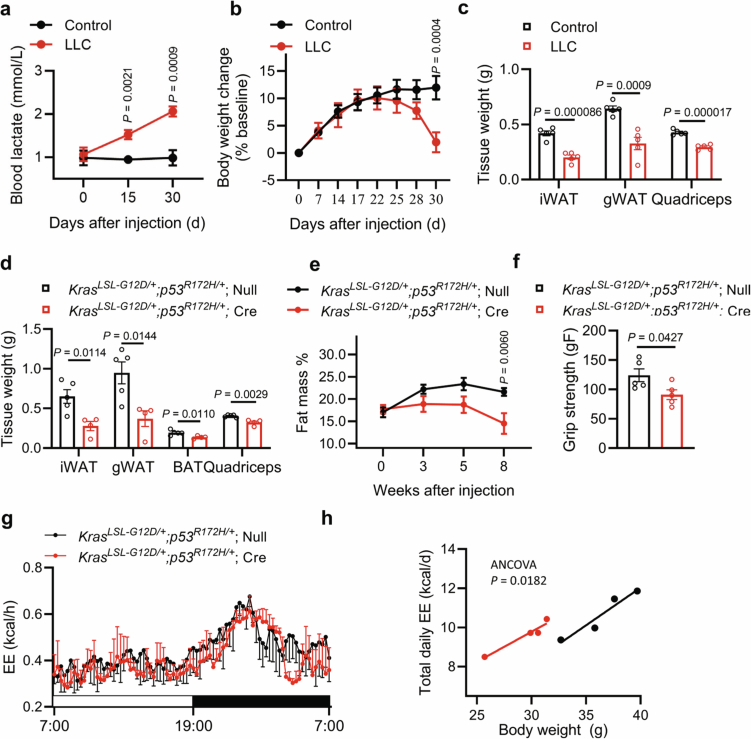
Cachectic phenotypes in orthotopic and genetically engineered mouse models of lung cancer. **a-c**, Orthotopic lung tumours induced an increase in blood lactate level (**a**), while decreases in body weight (**b**) and tissue mass of inguinal white adipose tissue (iWAT), gonad white adipose tissue (gWAT) and quadriceps (**c**) in mice (n = 5 for each group). **d-f**, Changes in tissue weights of iWAT, gWAT, brown adipose tissue (BAT), quadriceps (**d**); body fat percentage (**e**); and grip strength (**f**) of *Kras*^*LSL-G12D/+*^;*p53*^*R172H/+*^ mice after intranasal administration of control adenovirus (*Kras*^*LSL-G12D/+*^;*p53*^*R172H/+*^;Null, n = 5) or adenovirus expressing Cre recombinase (*Kras*^*LSL-G12D/+*^;*p53*^*R172H/+*^;Cre, n = 4). **g, h**, Energy expenditure (EE) over 24 h (**g**) and regression-based analysis of total daily EE against body weight (**h**) of *Kras*^*LSL-G12D/+*^;*p53*^*R172H/+*^ mice 8 weeks after adenovirus administration (n = 4). All data are presented as the mean ± s.e.m. *P* values were determined by two-tailed unpaired Student’s *t*-test (a, c, d, f), two-way ANOVA (b, e), or ANCOVA (h). Source data

To profile the systemic metabolic changes associated with cachexia, we utilized a mouse xenograft model of Lewis lung cancer (LLC) cells. The mice with tumour burden exhibited marked weight loss with decreases in white adipose tissue (WAT) and skeletal muscle mass (Fig. 1a,b and Extended Data Fig. 1a). Notably, the decrease of inguinal WAT (iWAT) mass was detectable as early as 12 days after cancer cell implantation, preceding that of skeletal muscle (Fig. 1b). Untargeted metabolomics analysis of the sera showed that samples from control and tumour-bearing mice were distinctly clustered into two groups (Extended Data Fig. 1b). Among the differential metabolites, lactate displayed the most significant increase in this cachexia model (Fig. 1c), and the identity of the peak in the mass spectrum and its concentration were confirmed by comparing to the standard solution of lactate (Extended Data Fig. 1c–e). Importantly, circulating lactate levels increased progressively with tumour growth and displayed a robust correlation with the body weight change in the tumour-bearing mice (Extended Data Fig. 1f,g), which was not due to decreased lactate uptake by major organs (Extended Data Fig. 1h). Next, we established an orthotopic mouse model of lung cancer by injecting LLC cells via a tail vein and observed increased blood lactate level and decreased adipose and muscle mass in the mice with lung tumours (Extended Data Fig. 2a–c). We tracked the changes in lactate levels, which occurred before a notable decrease in body weight (Extended Data Fig. 2a,b). To better model cancer-associated cachexia in patients, we generated a more clinically relevant mouse model of spontaneous lung cancer using *Kras*^*LSL-G12D/+*^;*p53*^*R172H/+*^ mice subjected to intranasal administration of adenovirus expressing Cre recombinase (*Kras*^*LSL-G12D/+*^;*p53*^*R172H/+*^;Cre)^5^. Notably, the *Kras*^*LSL-G12D/+*^;*p53*^*R172H/+*^;Cre mice experienced weight loss over weeks of cancer development (Fig. 1d), accompanied by a steady elevation in blood lactate level (Fig. 1e). Eight weeks after adenoviral Cre administration, *Kras*^*LSL-G12D/+*^;*p53*^*R172H/+*^;Cre mice had visible tumour nodules in the lungs (Fig. 1f). In addition, they exhibited the typical cachectic phenotype, including reduced tissue mass of WAT, brown adipose tissue (BAT) and skeletal muscle, weakened grip strength and augmented energy expenditure (EE) compared to the tumour-free controls (Extended Data Fig. 2d–h). Importantly, the blood lactate level is also closely correlated with body weight change in this genetically engineered mouse model of cancer cachexia (Fig. 1g).

**Extended Data Fig. 3:**
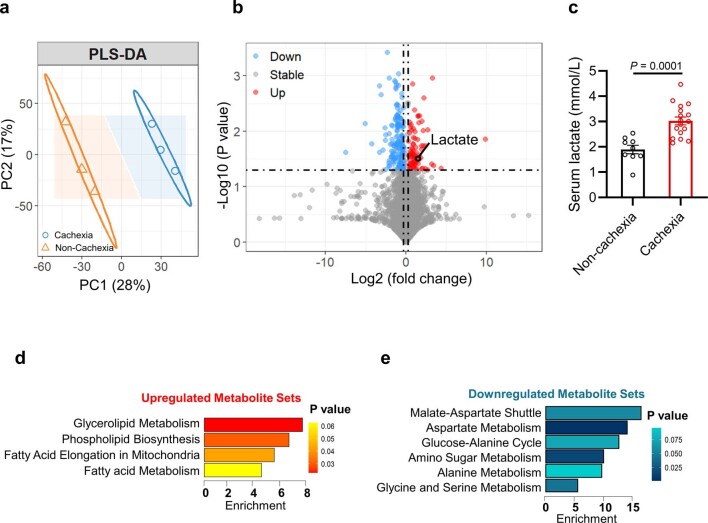
Differential serum metabolites in the patients with versus without cancer cachexia. **a**, PLS-DA of untargeted metabolomics profiling of serum samples from lung adenocarcinoma patients with or without cachexia (Cachexia and Non-cachexia, respectively; n = 3 for each group). **b**, Volcano plot showing the differential metabolites in the serum samples from patients with or without cancer cachexia. Metabolites with log2(fold change) ≥ 0.5 or ≤ 0.5 and *P* value < 0.05 were considered significantly different. Lactate, -log10(P value) = 1.49, log2(fold change) = 1.54. **c**, The serum lactate level in patients with or without cancer cachexia (Non-cachexia, n = 9; Cachexia, n = 17). **d,e**, The KEGG pathways enriched by analyzing the differential metabolites in the serum samples from cancer patients. All data are presented as the mean ± s.e.m. In panel c, *P* values were determined by two-tailed unpaired Student’s *t*-test. Source data

To investigate the potential clinical relevance of lactate in human cancer cachexia, serum samples were collected from patients with lung adenocarcinoma (Supplementary Table 1). Principal component analysis of the serum metabolites grouped the samples from patients with cachexia apart from those without cachexia (Extended Data Fig. 3a). Serum lactate level was also markedly elevated in the patients with cachexia (Extended Data Fig. 3b,c) and closely correlated with their body weight loss (Fig. 1h), substantiating the importance of lactate in the progression of cachexia. We noticed that the lactate level in patients dropped markedly after surgical removal of their lung tumours, suggesting that tumour is the major cause of the elevated serum lactate (Fig. 1i and Supplementary Table 2). In addition, the upregulated metabolites were enriched in glycerol and fatty acid metabolism, and the downregulated ones were pertained to the metabolism of amino acids (Extended Data Fig. 3d,e), consistent with previous reports^6^.

**Extended Data Fig. 4:**
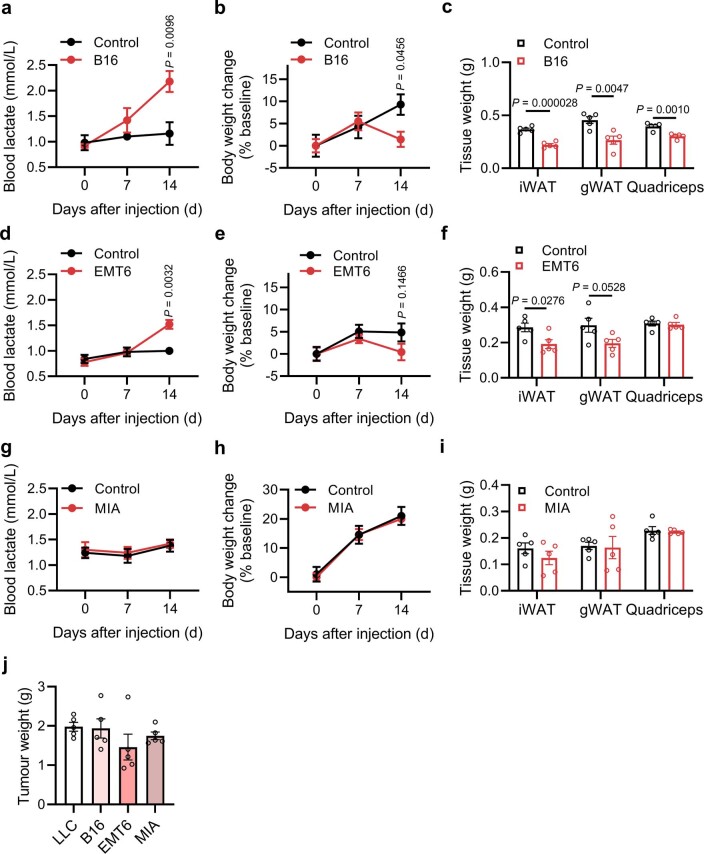
Lactate levels and body weight changes in mouse xenograft models. **a-c**, Blood lactate level (**a**), body weight (**b**), and tissue weights of inguinal white adipose tissue (iWAT), gonad white adipose tissue (gWAT) and quadriceps (**c**) of tumour-free (Control) and B16-F10 (B16) xenograft mice (n = 5 for each group). **d-f**, Blood lactate level (**d**), body weight (**e**), and tissue weights (**f**) of Control and EMT6 xenograft mice (n = 5 for each group). **g-i**, Blood lactate level (**g**), body weight (**h**), and tissue weights (**i**) of Control and MIA PaCa-2 xenograft (MIA) mice (n = 5 for each group). **j**, Weights of xenograft tumours derived from different cancer cells (n = 5 for each group). All data are presented as the mean ± s.e.m. *P* values were determined by two-tailed unpaired Student’s *t*-test (a, c, d, f, g, i), two-way ANOVA (b, e, h), or one-way ANOVA with Tukey’s multiple comparisons test (j). Source data

To examine whether this is true in other types of cancer, we subcutaneously xenoplanted mouse melanoma B16-F10 (B16), mouse mammary carcinoma EMT6 and human pancreatic carcinoma MIA PaCa-2 (MIA) cells into C57BL/6J, BALB/c and BALB/c nude mice, respectively. Among the three models tested, mice bearing B16 tumours displayed the largest increase in blood lactate and decrease in body weight (about 10%), along with substantial reductions in both WAT and muscle mass on day 14 (Extended Data Fig. 4a–c). Mice injected with EMT6 cells exhibited a moderate elevation of blood lactate, which was associated with a relatively milder cachectic phenotype (Extended Data Fig. 4d–f). However, there was no change in the lactate level, nor loss of body weight or WAT or muscle mass 2 weeks after injection of MIA cells (Extended Data Fig. 4g–i), although the tumour weights were about the same as those derived from the other cancer cell lines tested (Extended Data Fig. 4j). Thus, in all animal models examined and in patients with lung adenocarcinoma, tumour-induced cachexia was accompanied by an increase in circulating lactate.

## Increased blood lactate is sufficient to induce the cachectic phenotype

**Extended Data Fig. 5:**
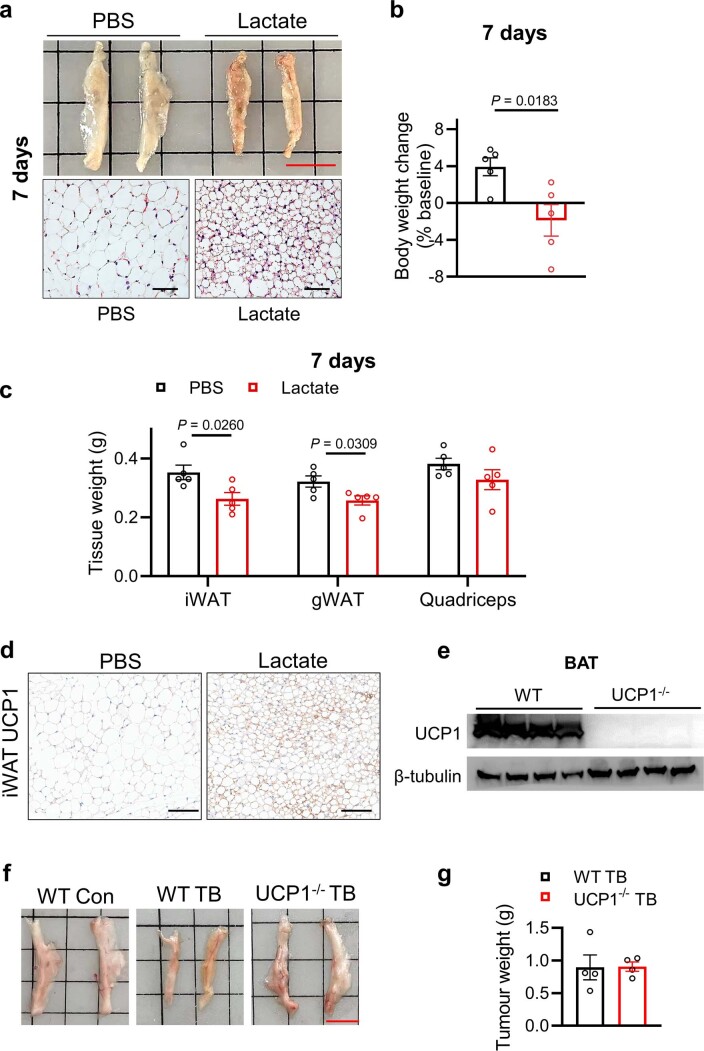
Lactate induces cachectic phenotype in mice, while UCP1 ablation ameliorates LLC-induced cachexia. **a**, Representative anatomical images and hematoxylin and eosin (H&E) staining of inguinal white adipose tissue (iWAT) from mice implanted with osmotic minipumps loaded with PBS or sodium L-lactate for 7 days. Scale bars, 1 cm in anatomical images (red bar in upper panel) and 100 μm in H&E staining (black bars in lower panels). **b,c**, Body weight (**b**) and tissue weights (**c**) of mice 7 days after implanted with osmotic minipumps loaded with PBS or sodium L-lactate (n = 5 for each group). **d**, Representative UCP1 immunohistochemical staining of the iWAT from mice 7 days after implanted with osmotic minipumps loaded with PBS or sodium L-lactate (n = 5 for each group). Scale bars, 100 μm. **e**, Representative western blots showing UCP1 protein level in the brown adipose tissue (BAT) from WT and UCP1 knockout (UCP1^−/−^) mice (n = 4). **f**, Representative anatomical images of iWAT from WT control, WT tumour-bearing (TB) and UCP1^-/-^ TB mice. Scale bar, 1 cm. **g**, Weight of tumours derived from WT TB and UCP1^-/-^ TB mice (n = 4). All data are presented as the mean ± s.e.m. In panel d, *P* value was determined by two-tailed unpaired Student’s *t*-test (b, c, g). Source data

To determine whether lactate plays a causal role in triggering cachexia, we utilized an osmotic minipump to clamp mouse blood lactate level at 1.5 mmol l^−1^ or 2 mmol l^−1^ (Fig. 1j), which was equivalent to the 50% increase in the patients with cachexia (Extended Data Fig. 3c) or matching the 100% increase in the LLC tumour-bearing cachectic mice (Fig. 1e and Extended Data Figs. 2a and 4a), respectively. After a 7-day infusion, l-lactate caused losses in body weight as well as WAT in a dose-dependent manner, but d-lactate had no effect (Fig. 1k,l and Extended Data Fig. 5a–c), indicating that l-lactate per se is sufficient to induce body weight decrease and adipose wasting. The failure of sodium d-lactate to trigger the cachexia phenotype excluded the potential unspecific effects caused by changes in pH, sodium concentration, or osmolality. Moreover, a prolonged l-lactate infusion lasting up to 14 days further decreased body weight as well as WAT mass (Fig. 1m–o). In particular, the wasting of skeletal muscle could also be observed after a 14-day lactate infusion (Fig. 1o). Taken together, these results indicate that lactate incites similar adipose remodelling as that induced by cancer cells. Therefore, lactate is not merely a characteristic metabolite in cancer cachexia, but also a key mediator of the cancer-induced hypercatabolic phenotype.

## Lactate induces cancer cachexia via GPR81

**Fig. 2:**
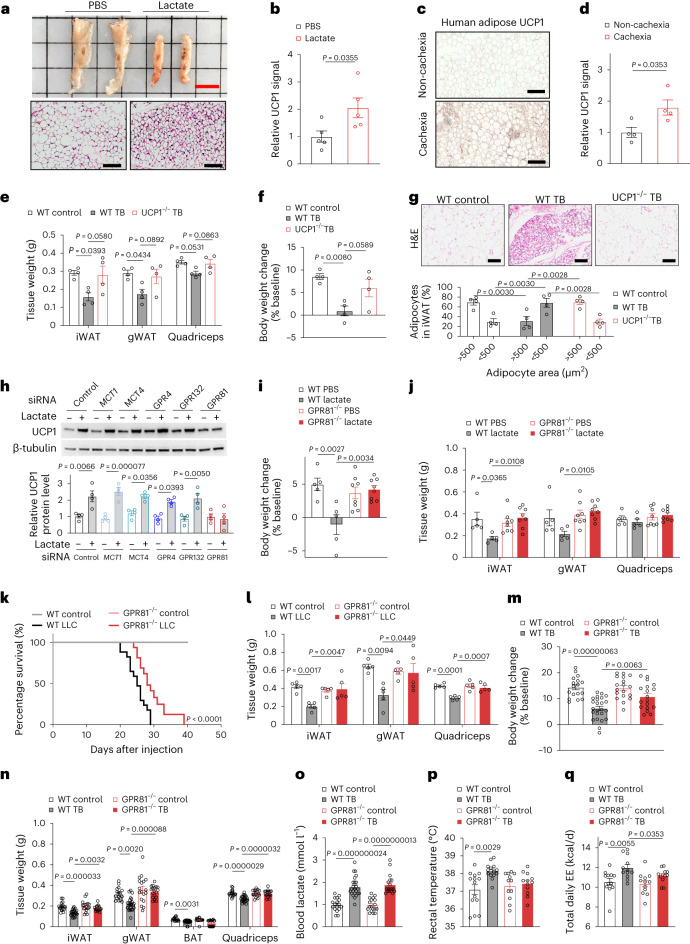
GPR81-deficient mice are resistant to lactate or tumour-induced cachexia. **a**, Representative anatomical image and H&E stainings of iWAT (*n* = 5 for each group). Scale bars, 1 cm (anatomical image) and 100 μm (H&E stainings). **b**, Statistic results of the signal intensity of UCP1 immunohistochemical staining in mouse iWAT (*n* = 5 for each group). **c**,**d**, Representative images (**c**) and statistical results (**d**) of adipose UCP1 immunohistochemical staining of patients (*n* = 4 for each group). Scale bars, 200 μm. **e**,**f**, Tissue weights (**e**) and body weight change (**f**) of WT control, WT tumour-bearing (WT TB) and UCP1 knockout (UCP1^−/−^) TB mice (*n* = 4 for each group). **g**, Representative images of H&E staining of iWAT and statistic results of cell sizes (*n* = 4 for each group). **h**, UCP1 protein levels in the SVF-derived adipocytes (*n* = 4 for each group). **i**,**j**, Body weights (**i**) and tissue weights (**j**) of the WT and GPR81 knockout (GPR81^−/−^) mice (WT, *n* = 5; GPR81^−/−^, *n* = 8). **k**, GPR81 depletion extended the survival of mice with orthotopic LLC tumours (WT control, *n* = 10; WT LLC, *n* = 16; GPR81^−/−^ control, *n* = 10; GPR81^−/−^ LLC, *n* = 13, *P* = 0.00000000000012). **l**, Tissue weights of WT and GPR81^−/−^ mice (*n* = 5 for each group). **m**–**o**, Body (**m**) and tissue weights (**n**) and blood lactate levels (**o**) of the WT and GPR81^−/−^ mice. (WT control, *n* = 17; WT TB, *n* = 24; GPR81^−/−^ control, *n* = 18; GPR81^−/−^ TB, *n* = 18). **p**, Rectal temperature of WT and GPR81^−/−^ mice with or without tumour. (WT, *n* = 14; GPR81^−/−^, *n* = 12 for each group). **q**, Total daily energy expenditure (EE) of WT and GPR81^−/−^ mice with or without tumours (*n* = 12 for each group). All data are presented as the mean ± s.e.m. *P* values were determined by two-tailed unpaired Student’s *t*-test (**b** and **d**), one-way ANOVA with Tukey’s multiple-comparisons test (**e**, **f**, **h**–**j** and **l**–**q**), simple survival analysis (Kaplan–Meier; **k**) and two-way ANOVA (**g**). Source data

Upregulation of adipose mitochondrial uncoupling protein 1 (UCP1), the marker of WAT browning, is a key feature in cachexia^7,8^. Lactate infusion also enhanced adipose UCP1 expression in mice assessed by immunohistochemical staining (Fig. 2a,b and Extended Data Fig. 5d), recapitulating adipose UCP1 upregulation in the patients with cancer-associated cachexia (Fig. 2c,d). More importantly, UCP1 ablation largely ameliorated LLC tumour-induced cachectic manifestations in the UCP1 knockout mice relative to their wild-type (WT) littermates with similar tumour burden (Fig. 2e–g and Extended Data Fig. 5e–g), suggesting that upregulation of UCP1 is essentially involved in the pathogenesis of cancer cachexia.

**Extended Data Fig. 6:**
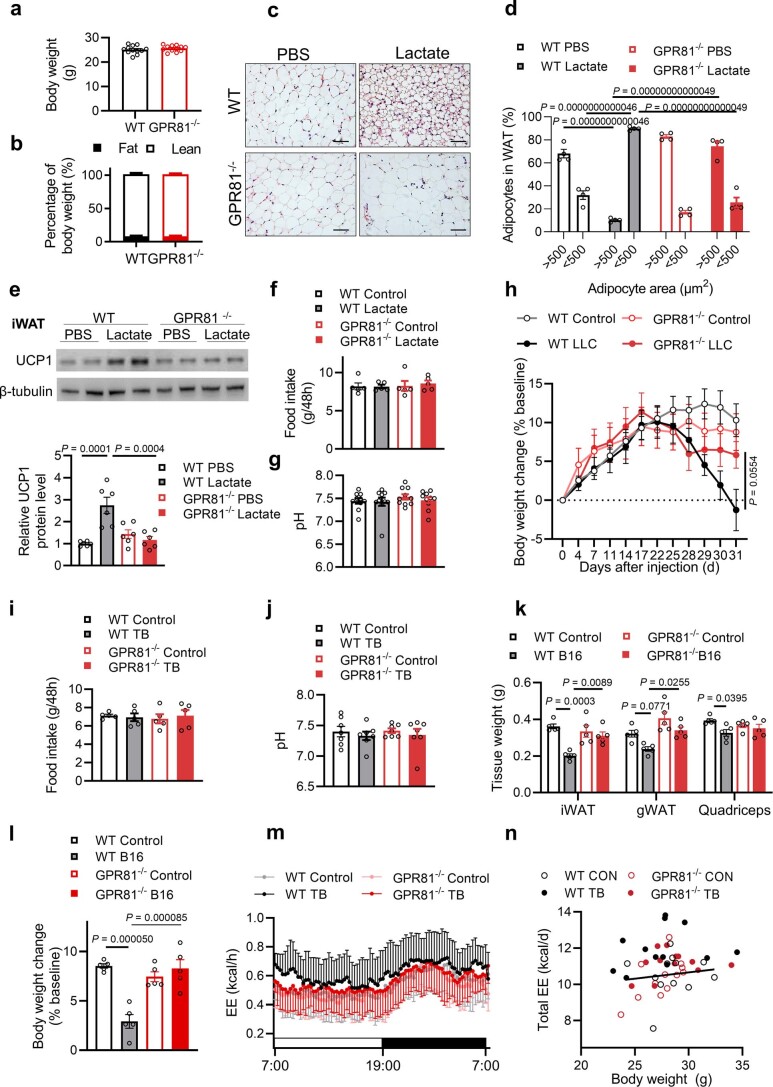
GPR81 ablation ameliorates lactate- or tumour-induced cachexia in mouse models. **a,b**, Body weight (**a**) and body composition (**b**) of 7-week-old GPR81 knockout (GPR81^-/-^) mice and their wild type (WT) littermates (n = 12 for each group). **c, d**, Hematoxylin and eosin (H&E) staining (**c**) and statistic results of the distribution of adipocyte sizes (**d**) in the inguinal white adipose tissue (iWAT) from WT and GPR81^-/-^ mice 7 days after implanted with osmotic minipumps loaded with PBS or sodium L-lactate (n = 4 for each group). In panel **c**, the images of WT iWAT were the same as those in the Extended Data Fig. 5a, as they were from the same experiment. Scale bars, 100 μM. **e**, Representative western blots and statistic data showing UCP1 protein level in the iWAT from WT and GPR81^-/-^ mice 7 days after implanted with osmotic minipumps loaded with PBS or sodium L-lactate (n = 5 for each group). **f, g**, The food intake (**f**, n = 5 for each group) and pH of blood (**g**, n = 10 for each group) in the mice implanted with lactate minipumps. **h**, Body weight of WT and GPR81^-/-^ mice with orthotopic lung cancer (n = 5 for each group). **i, j**, The food intake (**i**, n = 5 for each group) and pH of blood (**j**, n = 7 for each group) of WT and GPR81^-/-^ mice with LLC xenograft tumours. **k, l**, Tissue weights of iWAT, gonad white adipose tissue (gWAT), quadriceps (**k**) and body weight change (**l**) of WT and GPR81^-/-^ mice xenoplanted with B16-F10 cells. (n = 5 for each group). **m, n**, Energy expenditure (EE) over 24 h (**m**) and regression-based analysis of total daily EE against body mass (**n**) of WT and GPR81^-/-^ mice 24 days after xenoplanted with LLC cells subcutaneously. (Control, tumour-free mice; TB, mice xenograft with LLC cells; n = 12 for each group). All data are presented as the mean ± s.e.m. *P* values were determined by two-tailed unpaired Student’s *t*-test (a, b, h), two-way ANOVA (d), or one-way ANOVA with Tukey’s multiple comparisons test (e-g, i-l), or ANCOVA (n). Source data

To identify the sensor(s) for lactate in adipocytes, we individually knocked down the expression of lactate transporter monocarboxylate transporter 1 and 4 (MCT1 and MCT4, respectively)^9^, as well as its known receptors such as GPR132 (ref. ^10^), GPR4 (ref. ^11^) or GPR81 (ref. ^12^) in adipocytes derived from adipose stromal vascular fraction (SVF). Remarkably, lactate-induced upregulation of UCP1 was abolished only by GPR81-specific siRNA but not the siRNAs targeting other candidates (Fig. 2h). To further explore the function of GPR81 in cachexia, we utilized GPR81 knockout (GPR81^−/−^) mice^12^ whose body weight and body composition were comparable to their WT littermates at baseline (Extended Data Fig. 6a,b). Seven days after lactate delivery via minipump, there was no decline in body weight or change in adipose tissue mass, cell size or UCP1 expression in the GPR81^−/−^ mice (Fig. 2i,j and Extended Data Fig. 6c–e), and the pH of blood or food intake was not affected by lactate infusion or GPR81 depletion (Extended Data Fig. 6f,g). In the orthotopic lung cancer model, GPR81 ablation markedly extended the lifespan of the tumour-bearing GPR81^−/−^ mice (Fig. 2k), besides ameliorating the cancer-associated cachectic phenotype (Fig. 2l and Extended Data Fig. 6h). In the LLC xenograft model, the GPR81^−/−^ mice maintained body weight as well as fat and muscle mass, although tumour-induced elevation of blood lactate was comparable to that in the WT littermates (Fig. 2m–o), and this increase in lactate level did not cause acidemia or anorexia (Extended Data Fig. 6i,j). GPR81 depletion also blocked B16 tumour-induced weight loss (Extended Data Fig. 6k,l). Furthermore, GPR81 deficiency abolished LLC tumour-triggered low-grade fever (a common clinical manifestation of cancer cachexia; Fig. 2p) and increases in EE (Fig. 2q and Extended Data Fig. 6m,n).

**Fig. 3:**
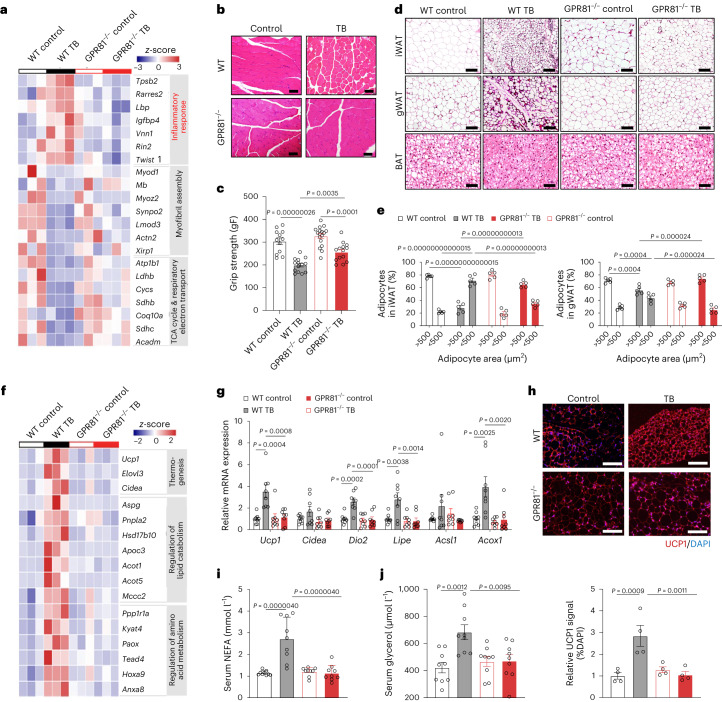
GPR81 ablation mitigates the tumour-induced wasting of muscle and adipose tissue. **a**, Heat map illustrating the differential gene expression profiles of skeletal muscle of WT and GPR81 knockout (GPR81^−/−^) mice with or without subcutaneously xenograft LLC tumours (*n* = 3 for each group). **b**,**c**, Representative images of H&E staining (**b**) and grip strength (**c**) of skeletal muscle of WT control (*n* = 12), WT TB (*n* = 14), GPR81^−/−^ control (*n* = 17) and GPR81^−/−^ tumour-bearing (GPR81^−/−^ TB, *n* = 14). Scale bars, 100 μm in **b**. **d**, Representative images of H&E staining of iWAT, gWAT and BAT. *n* = 5 for each group. Scale bars, 100 μm. **e**, Statistic results showing the distribution of adipocyte sizes in the iWAT and gWAT from WT and GPR81^−/−^ mice with or without tumour (*n* = 5 for each group). **f**, Heat map illustrating the differential gene expression profiles of iWAT (*n* = 3 for each group). **g**, Relative mRNA levels of thermogenesis-related genes in the iWAT determined by quantitative PCR with reverse transcription (RT–qPCR; *n* = 8 for each group). **h**, Representative images and statistical results of immunofluorescence staining of UCP1 (red) in iWAT from WT and GPR81^−/−^ with or without tumour (*n* = 4 for each group). Nuclei were stained with DAPI (blue). Scale bars, 100 μm. **i**,**j**, Serum NEFA (**i**) and glycerol (**j**) levels of WT and GPR81^−/−^ mice with or without tumour (*n* = 9). All data are presented as the mean ± s.e.m. *P* values were determined by one-way ANOVA with Tukey’s multiple-comparisons test (**c**, **g**–**j**) or two-way ANOVA (**e**). Source data

Skeletal muscle atrophy and weakness are the key clinical symptoms of cancer cachexia. Transcriptome analysis of skeletal muscle in the LLC xenograft model showed that cancer upregulated genes enriched in inflammation responses, but it repressed genes involved in myofibril assembly and muscle cell development, as well as the tricarboxylic acid cycle and generation of precursor metabolites and energy (Fig. 3a). These changes were all markedly mitigated by GPR81 ablation. As a result, the skeletal muscle structure was better preserved, and the grip strength was noticeably improved in the GPR81^−/−^ tumour-bearing mice compared to their WT littermates (Fig. 3b,c).

**Extended Data Fig. 7:**
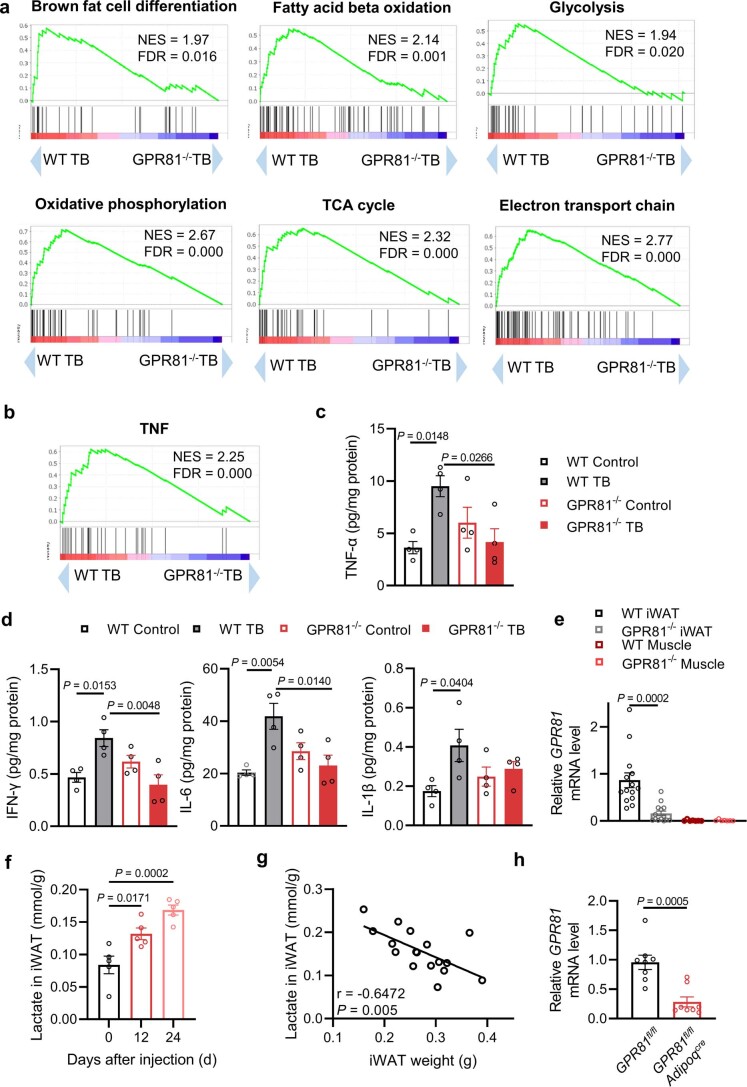
Adipose GPR81 plays an important role in tumour-induced WAT browning and inflammation. **a**, GSEA identified WAT browning-related gene sets were significantly down-regulated in the inguinal white adipose tissue (iWAT) of GPR81 knockout tumour-bearing (GPR81^-/-^ TB) relative to the wild type tumour-bearing (WT TB) mice. (n = 3 for each group). **b**, TNF signaling-related gene set was down-regulated in the iWAT of GPR81^-/-^ TB as compared with WT TB (n = 3 for each group). **c,d**, The levels of TNF-α (**c**), IFN-γ, IL-6, and IL-1β (**d**) in the iWAT assessed by ELISA (n = 4 for each group). **e**, Relative *GPR81* mRNA levels in the iWAT from WT and GPR81^-/-^ mice (n = 14 for each group) and their quadriceps (n = 7 for each group). **f**, Lactate levels of the iWAT interstitial fluid at baseline, 12 and 24 days after xenoplanted with LLC cells subcutaneously (n = 5 for each group). **g**, Linear regression analysis showing the correlation between the iWAT weight with iWAT interstitial fluid lactate level 24 days after xenoplanted with LLC cells (n = 17). **h**, Statistic results showing *GPR81* mRNA levels in the iWAT from *GPR81*^fl/fl^ and *GPR81*^fl/fl^*Adipoq*^*cre*^ mice (n = 8 for each group). All data are presented as the mean ± s.e.m. *P* values were determined by one-way ANOVA with Tukey’s multiple comparisons test (c, d, f) and two-tailed unpaired Student’s *t*-test (e). *P* value of the correlation was determined by two-tailed Pearson correlation analysis (g). Source data

The WT tumour-bearing mice exhibited severe adipose tissue wasting, manifested by smaller adipocytes in iWAT and gonadal white adipose tissue (gWAT) depots and shrinkage of lipid droplets in BAT (Fig. 3d). In WT mice, the percentage of adipocytes with an area larger than 500 µm^2^ dropped from about 80% to ~30% in iWAT and ~55% in gWAT after tumour implantation, whereas the sizes of cells and lipid droplets in the GPR81^−/−^ tumour-bearing mice were unaffected (Fig. 3d,e). KEGG (Kyoto Encyclopedia of Genes and Genomes) analysis revealed that genes of thermogenesis as well as lipid and amino acid catabolism were increased in the iWAT of WT but not GPR81^−/−^ tumour-bearing mice (Fig. 3f,g). Remarkably, GPR81 deficiency blocked tumour-induced UCP1 expression, indicating suppressed iWAT browning (Fig. 3h). Furthermore, Gene Set Enrichment Analysis (GSEA) profiling of RNA-sequencing (RNA-seq) data demonstrated that browning-related and lipolysis-related gene sets, including brown fat cell differentiation, fatty acid oxidation, glycolysis, oxidative phosphorylation, tricarboxylic acid cycle and electron transport chain, were systematically downregulated in the iWAT of tumour-bearing GPR81^−/−^ relative to WT mice (Extended Data Fig. 7a). Concomitantly, the WT tumour-bearing mice had marked elevations of circulating non-esterified fatty acids (NEFAs) and glycerol, which was not observed in the GPR81^−/−^ mice (Fig. 3i,j). In addition to the correction of metabolic reprogramming, adipose inflammation was suppressed by GPR81 deletion, manifested by decreased expression of genes related to TNF signalling, such as TNF-α (Extended Data Fig. 7b,c) and several other inflammatory cytokines, including IFN-γ, IL-6 and IL-1β (Extended Data Fig. 7d). Taken together, GPR81 mediates the catabolic effects of lactate, whereas GPR81 ablation markedly mitigates the cachectic manifestations induced by lactate and tumour.

## Role of adipose tissue in the development of cancer cachexia

**Fig. 4:**
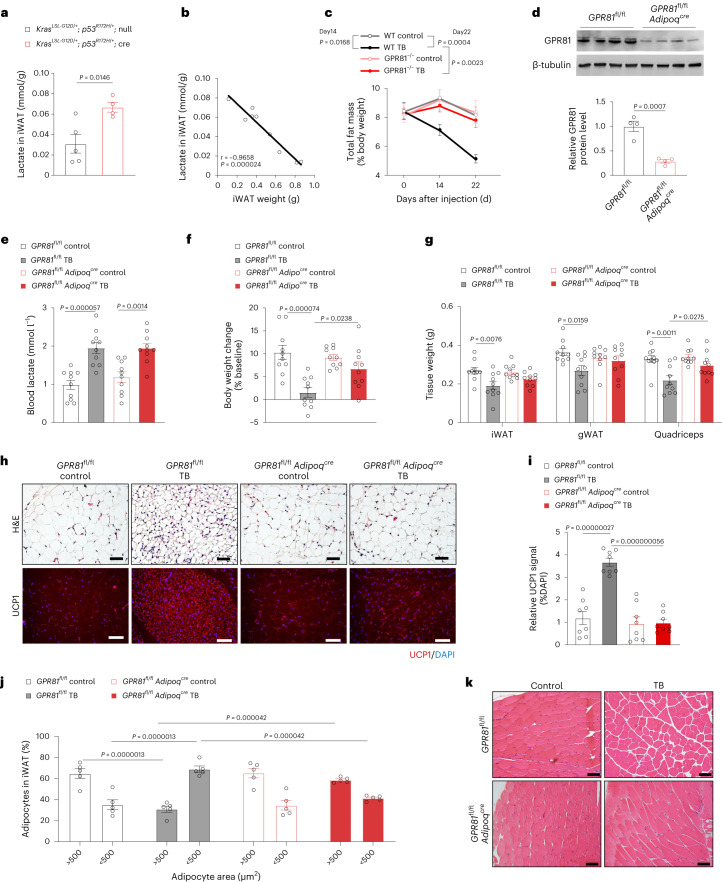
Adipose GPR81 is the major mediator of pro-catabolic effects of lactate. **a**, Lactate levels of the interstitial fluid of iWAT from *Kras*^*LSL-G12D/+*^;*p53*^*R172H/+*^ mice after intranasal administration of control adenovirus (*n* = 5) or adenovirus expressing Cre recombinase (*n* = 4). **b**, Linear regression analysis showing the correlation between iWAT weights and lactate levels of the iWAT interstitial fluid from *Kras*^*LSL-G12D/+*^;*p53*^*R172H/+*^ mice after intranasal administration of adenovirus (*n* = 9). **c**, Change in the body fat percentage in the WT tumour-free (WT control, *n* = 15), WT TB (*n* = 14), GPR81 knockout tumour-free (GPR81^−/−^ control, *n* = 12) and GPR81^−/−^ TB (*n* = 15) mice determined by magnetic resonance imaging. **d**, Representative western blot and statistical data showing GPR81 protein levels in the iWAT isolated from *GPR81*^fl/fl^ and *GPR81*^fl/fl^*Adipoq*^*cre*^ mice (*n* = 4 for each group). **e**–**g**, Blood lactate levels (**e**), body weight change (**f**) and tissue weights (**g**) in the tumour-free (control) *GPR81*^fl/fl^ and *GPR81*^fl/fl^*Adipoq*^*cre*^ mice or implanted with LLC cells (TB) for 24 days (*n* = 10 for each group). **h**, Representative images of H&E staining and immunofluorescence staining of UCP1 in the iWAT from *GPR81*^fl/fl^ and *GPR81*^fl/fl^*Adipoq*^*cre*^ mice with or without LLC tumour (*n* = 5 for each group). Scale bars, 100 μm. **i**, Statistical results of the immunofluorescence signal intensity of UCP1 in the iWAT from *GPR81*^fl/fl^ and *GPR81*^fl/fl^*Adipoq*^*cre*^ mice with or without LLC tumour (*n* = 8 for each group). **j**, Statistical results showing the distribution of adipocyte sizes in the iWAT from *GPR81*^fl/fl^ and *GPR81*^fl/fl^*Adipoq*^*cre*^ mice with or without tumour (*n* = 5 for each group). **k**, Representative images of H&E staining of skeletal muscle from *GPR81*^fl/fl^ and *GPR81*^fl/fl^*Adipoq*^*cre*^ mice with or without tumour (*n* = 5 for each group). Scale bars, 100 μm. All data are presented as the mean ± s.e.m. *P* values were determined by two-tailed unpaired Student’s *t*-test (**a** and **d**), two-way ANOVA (**c** and **j**) and one-way ANOVA (**e**–**g** and **i**). *P* value of the correlation was determined by two-tailed Pearson correlation analysis (**b**). Source data

GPR81 was highly expressed in the adipose tissue (Extended Data Fig. 7e) as reported previously^12,13^, implying that adipose GPR81 signalling may be of particular importance in the development of cancer cachexia. Indeed, iWAT interstitial fluid lactate level was not only increased in the tumour-bearing mice (Fig. 4a and Extended Data Fig. 7f), but also negatively correlated with the iWAT weight in the *Kras*^*LSL-G12D/+*^;*p53*^*R172H/+*^;Cre mice and LLC xenograft model (Fig. 4b and Extended Data Fig. 7g). The decrease of iWAT mass preceded that of skeletal muscle (Fig. 1b) and the decrease of body fat percentage was blocked by GPR81 deficiency (Fig. 4c), suggesting that the GPR81-dependent maladaptation of adipose tissue is an early event in the pathogenesis of cancer cachexia.

To nail down the role of adipose GPR81 in the pathogenesis of cachexia, we generated adipose tissue-specific GPR81 knockout mice (*GPR81*^fl/fl^*Adipoq*^*cre*^) that had about 70% reduction of GPR81 expression at its protein and mRNA levels in adipocytes (Fig. 4d and Extended Data Fig. 7h). Xenograft of LLC cells elevated blood lactate levels equally regardless of adipose GPR81 expression level (Fig. 4e). However, tumour-induced body weight loss as well as WAT and skeletal muscle remodelling were all attenuated by the depletion of adipose GPR81 (Fig. 4f–k). These data indicate that adipose GPR81 is essential for the development of cancer cachexia.

## Lactate induces WAT browning via GPR81–Gi–Gβγ–RhoA/ROCK1–p38 cascade

**Fig. 5:**
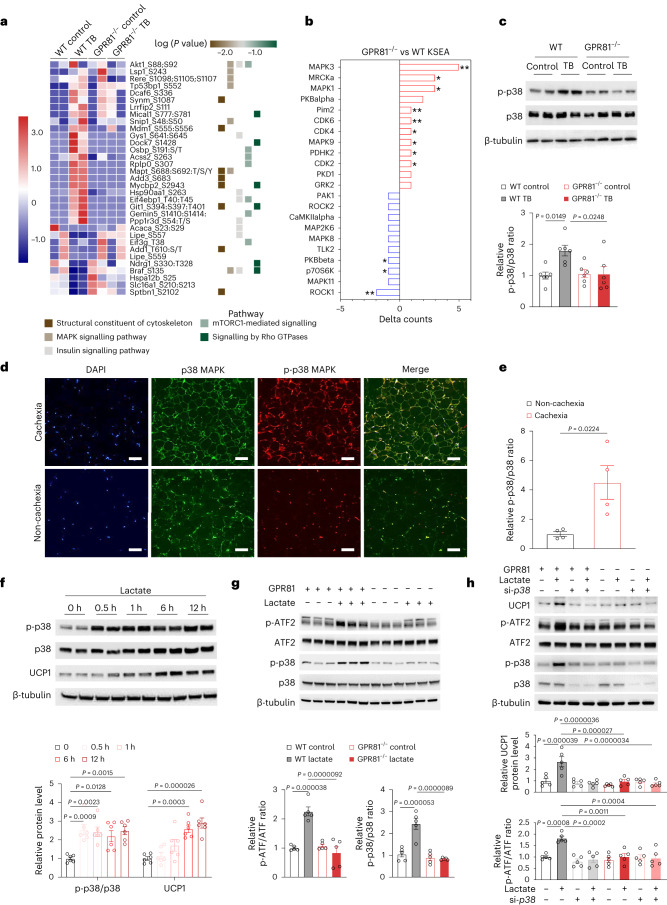
GPR81 induces WAT browning through activation of p38. **a**, Heat map showing the differentially phosphorylated proteins and the enriched pathways in the iWAT from WT tumour-free (WT control), WT TB, GPR81 knockout tumour-free (GPR81^−/−^ control) and GPR81^−/−^ tumour-bearing (GPR81^−/−^ TB) mice. **b**, Result of Kinase–Substrate Enrichment Analysis (KSEA) showing changes in kinase activity in the iWAT of tumour-bearing mice due to GPR81 ablation. Delta counts indicate the number of substrates of each kinase in the iWAT of GPR81^−/−^ TB minus that in the WT TB mice. **c**, Representative western blots and statistical data showing the levels of phosphorylated (p-p38) and total p38 in the iWAT (*n* = 6 for each group). **d**,**e**, Representative images (**d**) and statistical data (**e**) of immunofluorescence signal intensity of p38 (green) and phosphorylated p38 (p-p38; red) in the adipose tissue from patients with or without cancer cachexia (*n* = 4 for each group). The nuclei were stained with DAPI (blue). Scale bars, 100 μm. **f**, Representative western blots and statistical data showing the levels of phosphorylated (p-p38), total p38 and UCP1 in the SVF-derived adipocytes treated with sodium l-lactate for the indicated time points (*n* = 6 biologically independent samples in each group). **g**, Representative western blots and statistical data showing the lactate-induced activation of p38-ATF2 was abolished in GPR81^−/−^ SVF-derived adipocytes (*n* = 5 biologically independent samples in each group). **h**, Knocking down the expression of *p38* by specific siRNA abolished the lactate-induced activation of p38-ATF2 and upregulation of UCP1 (*n* = 5 biologically independent samples in each group). All data are presented as the mean ± s.e.m. *P* values were determined by one-way ANOVA with Tukey’s multiple-comparisons test (**c** and **f**–**h**) and two-tailed unpaired Student’s *t*-test (**e**). In **b**, KSEA was used to analyze the possible kinases of the differentially phosphorylated peptides and the statistical significance was calculated by hypergeometric test; *p < 0.05, ** *p* < 0.01. Source data

To delineate the signalling events mediating lactate/GPR81-induced metabolic remodelling of adipose tissue, we performed phosphoproteomics analysis of iWAT from the WT and GPR81^−/−^ mice with or without LLC-induced cachexia. The iWAT tissue samples from four mice were pooled together as one sample, and each group had two samples. The counts of phosphorylated peptides or proteins were similar among the four groups (Supplementary Fig. 1a–d). Principal component analysis was used to cluster the samples into three separate groups, that is, the tumour-free mice with or without GPR81, WT tumour-bearing and GPR81^−/−^ tumour-bearing mice (Supplementary Fig. 1e). KEGG analysis enriched pathways related to structure remodelling, insulin and mTOR signalling, mitogen activated protein kinases (MAPKs), and Rho GTPase-related pathways (Fig. 5a). Specifically, MAPK3 (ERK1), MAPK1 (ERK2), and MAPK9 (JNK2) were highly activated, while MAPK8 (JNK1) and MAPK11 (p38) were markedly repressed in the GPR81^−/−^ iWAT (Fig. 5b,c). These results suggest that GPR81 deficiency may block tumour-induced iWAT wasting by preserving the ERK1/2-mediated adipogenic programme^14^ while inhibiting the p38-promoted browning programme^15–18^. In line with the observation in mice, p38 was activated in the iWAT of patients with cachexia induced by lung adenocarcinoma, as evidenced by enhanced immunofluorescence signal of phosphorylated p38 (Fig. 5d,e).

It has been shown that activated p38 facilitates nuclear translocation of activating transcription factor 2 (ATF2) by phosphorylation, leading to increased UCP1 expression^19^. In SVF-derived adipocytes, lactate treatment enhanced phosphorylation of p38 in a time-dependent manner (Fig. 5f), while GPR81 knockout blocked the lactate-induced activation of p38 and ATF2 (Fig. 5g). Notably, p38 deficiency by gene silencing repressed the lactate-induced upregulation of UCP1 (Fig. 5h), suggesting that the function of lactate/GPR81 is mediated by p38.

**Fig. 6:**
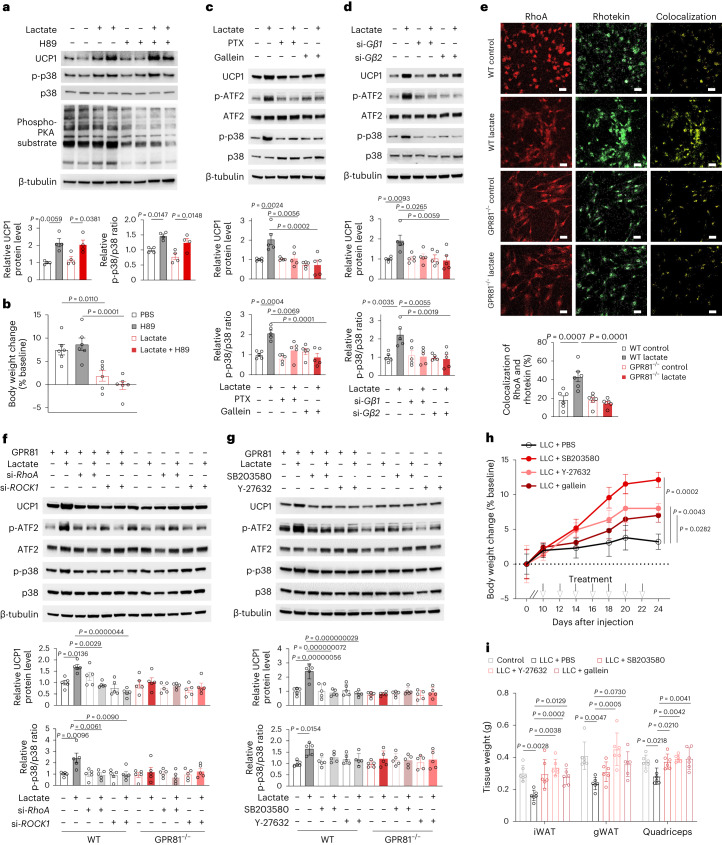
Activation of GPR81 induces WAT browning via Gβγ–RhoA–p38 cascade. **a**, Representative western blots and statistical results showing the levels of phospho-PKA substrates, phosphorylated p38 (p-p38) and UCP1 in the SVF-derived adipocytes treated with PKA inhibitor H89 (*n* = 4 biologically independent samples in each group). **b**, Body weights of mice implanted with osmotic minipumps loaded with PBS or sodium l-lactate and treated with H89 or vehicle (*n* = 6 for each group). **c**,**d**, Representative western blots and statistical results showing that the lactate-induced activation of p38-ATF2 and upregulation of UCP1 were blocked by inhibitor for Gαi (PTX) or Gβγ (gallein) (**c**) or silencing the expression of *Gβ1* or *Gβ2* by siRNA (**d**) (*n* = 5 biologically independent samples in each group). **e**, Representative images and statistical results of immunofluorescence staining of the SVF-derived adipocytes with antibodies against RhoA and rhotekin (*n* = 6 biologically independent samples in each group). Scale bars, 20 μm. **f**,**g**, Representative western blots and statistical results showing that knocking down the expression of *RhoA* or *ROCK1* by siRNA (**f**) or treating the cells with inhibitor for p38 (SB203580) or ROCK1 (Y-27632) (**g**) abolished the lactate-induced activation of p38-ATF2 and upregulation of UCP1 (*n* = 5 biologically independent samples in each group). **h**,**i**, Injection of inhibitor for p38 (SB203580), ROCK1 (Y-27632) or Gβγ (gallein) into the inguinal fat pads in the tumour-bearing mice alleviated tumour-induced losses of body weight (**h**) and tissue weights (**i**) (*n* = 6 for each group). All data are presented as the mean ± s.e.m. *P* values were determined by one-way ANOVA with Tukey’s multiple-comparisons test (**a**–**g** and **i**), or two-tailed unpaired Student’s *t*-test comparing body weights 24 days after injection of LLC cells (**h**). Source data

**Extended Data Fig. 8:**
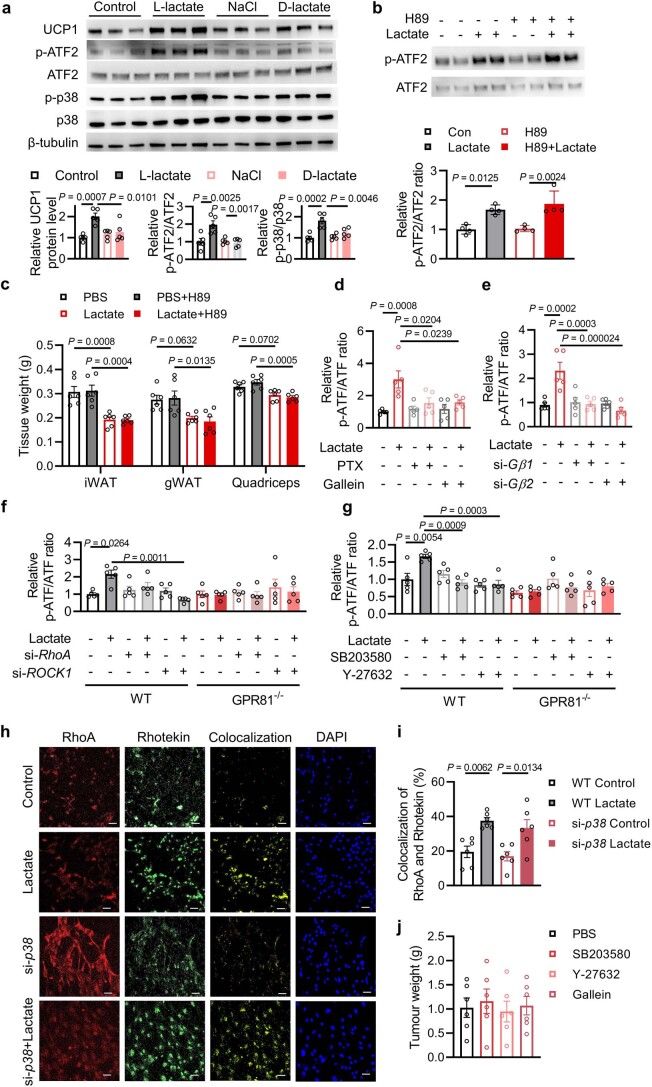
Inhibition of p38, ROCK1, or Gβγ ameliorates lactate-induced browning in adipocytes. **a**, Representative western blots and statistic results of UCP1 and activation of p38-ATF2 in the SVF-derived adipocytes after treatment with 20 mM sodium L-lactate, NaCl, or sodium D-lactate (n = 6 biologically independent samples in each group). **b**, Representative western blots and ratio of phosphorylated to total ATF2 in the SVF-derived adipocytes, showing that lactate-induced activation of p38-ATF2 was not blocked by PKA inhibitor H89 (n = 4 biologically independent samples in each group). **c**, Tissue weights of inguinal white adipose tissue (iWAT), gonad white adipose tissue (gWAT), and quadriceps of mice implanted with osmotic pumps loaded with PBS or sodium L-lactate and treated with H89 (n = 6 for each group). **d-g**, Statistic results showing that in the SVF-derived adipocytes, treatment with inhibitor for Gαi (PTX) or Gβγ (Gallein) (**d**, n = 5 biologically independent samples in each group), silencing the expression of *Gβ1* or *Gβ2* by siRNA (**e**, n = 5 biologically independent samples in each group), silencing the expression of RhoA or ROCK1 by specific siRNA (**f**, n = 5 biologically independent samples in each group), or treatment with inhibitor for p38 (SB203580) or ROCK1 (Y-27632) (**g**, n = 5 biologically independent samples in each group) abolished the lactate-induced activation of ATF2. **h,i**, Immunofluorescence staining (**h**) and statistic result of the signal intensity (**i**) of colocalized RhoA and Rhotekin in the SVF-derived adipocytes (n = 6 biologically independent samples in each group). Scale bars, 20 μm. **j**, Weights of LLC tumours derived from mice injected in the inguinal fat pad with inhibitor for p38, ROCK1, or Gβγ (n = 6 for each group). All data are presented as the mean ± s.e.m. *P* values were determined by one-way ANOVA with Tukey’s multiple comparisons (a-g, i, j). Source data

Additionally, treating SVF-derived adipocytes with sodium l-lactate, but not sodium d-lactate or sodium chloride, led to markedly enhanced phosphorylation of p38 and activated its downstream signalling as evidenced by increased ATF2 phosphorylation and UCP1 expression (Extended Data Fig. 8a), indicating that the induction of thermogenic signalling is caused by l-lactate-mediated activation of GPR81 rather than by nonspecific response to changes in osmolarity or sodium concentration. Since multiple stimuli converge on PKA to stimulate thermogenesis^20^, we utilized PKA inhibitor H89 (ref. ^21^) to explore whether activation of PKA is involved in lactate/GPR81-mediated cachectic signalling. Inhibition of PKA by H89 failed to suppress lactate-induced UCP1 upregulation in vitro (Fig. 6a and Extended Data Fig. 8b) or body and adipose weight loss in vivo (Fig. 6b and Extended Data Fig. 8c), indicating that PKA is not required for lactate-activated cachectic signalling.

GPR81 is a Gαi/o-coupled receptor^12,13^. In SVF-derived adipocytes, lactate-induced p38 activation and subsequent adipose browning was blocked not only by ribosylation of Gαi/o with PTX (Fig. 6c), but also by a Gβγ inhibitor gallein or knocking down the expression of *Gβ1* or *Gβ2* (Fig. 6c,d and Extended Data Fig. 8d,e); suggesting an essential involvement of Gαi/o-Gβγ. Regarding the downstream signalling components, we provided several lines of evidence that RhoA/ROCK1 partakes the GPR81–p38 signalling cascade. First, the phosphoproteomics data showed that Rho GTPase-mediated signalling and RhoA effector kinase ROCK1 were repressed in the GPR81-deficient iWAT (Fig. 5a,b). Second, lactate promoted colocalization of RhoA with the Rho GTPase effector rhotekin in the SVF-derived adipocytes in a GPR81-dependent manner (Fig. 6e). More importantly, inhibiting the expression of *RhoA* or *ROCK1* by specific siRNA, or using a ROCK1 inhibitor Y-27632, markedly blunted the lactate-induced activation of p38 (Fig. 6f,g and Extended Data Fig. 8f,g). In contrast, suppressing *p38* expression did not affect lactate-induced RhoA activation (Extended Data Fig. 8h,i), indicating that activation of RhoA/ROCK1 is upstream of p38.

**Extended Data Fig. 9:**
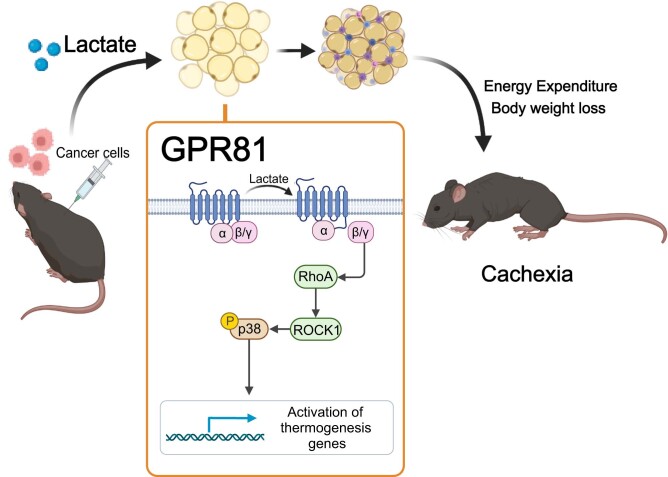
Schematic illustration depicting the signaling cascade downstream of lactate/GPR81 in promoting cancer cachexia. Activation of GPR81 by lactate instigates adipose tissue browning via Gαi/o-Gβγ-RhoA-ROCK1-p38 signaling cascade, which promotes the development of cachexia. Created with BioRender.com.

To validate the role of this signalling cascade in mediating the pro-cachectic effect of lactate, tumour-bearing mice were treated with gallein, Y-27632 or SB203580, the inhibitor of Gβγ, ROCK1 or p38, respectively, starting on the tenth day after xenograft with LLC cells. Remarkably, injecting each inhibitor in the inguinal fat pad did not influence tumour growth (Extended Data Fig. 8j) but effectively alleviated LLC-induced body weight and tissue weight loss in mice (Fig. 6h,i). Altogether, these results indicate that lactate-induced activation of GPR81 triggers WAT browning mainly via the Gβγ–RhoA/ROCK1–p38 signalling pathway (Extended Data Fig. 9).

## Discussion

In searching for the link connecting tumour to extensive catabolism in cancer cachexia, we have utilized unbiased metabolomics screening of a mouse model of cancer cachexia and identified lactate as the top differential metabolite whose level is tightly correlated with the reduction of body weight. This is also true for patients with lung adenocarcinoma who have cancer cachexia. Importantly, the elevation of circulating and adipose interstitial lactate occurs before body weight loss, and lactate infusion results in similar wasting phenotype as induced by tumour. Unlike administering lactate with a bolus injection that induces a transient change in sodium concentration and osmolarity^22^, lactate infusion via osmotic minipump resulted in a sustained moderate increase of circulating lactate without alteration in blood pH. Moreover, d-lactate supplied via minipump demonstrated no effect in weight loss, suggesting that the catabolic effects of l-lactate are not attributable to alterations in sodium concentration, osmolarity or pH. It is well established that cancer cells utilize aerobic glycolysis, a phenomenon called the Warburg effect, to support their rapid proliferation. The augmented production of lactate leads to a sustained increase in blood lactate level in many patients with cancer^23^, which is negatively associated with their prognosis^24^.

Among several known lactate sensors, we have pinpointed adipose GPR81 as the predominant mediator of the pro-catabolic effects of lactate because GPR81 deficiency blocks tumour-triggered as well as lactate infusion-triggered cachectic manifestations. In contrast, other potential candidates, including MCT1, MCT4, GPR4 or GPR132, have no contribution to the lactate-activated cachectic signalling. These findings have established lactate/GPR81 as the key link between tumour and metabolic reprogramming in cancer cachexia. The results of this study also demonstrate the catabolic remodelling of WAT as the early pathological event in cancer cachexia, although skeletal muscle atrophy is a major contributor of impaired physical functioning in patients with cachexia^25^. In line with our observations, body fat loss can occur in the absence of a decrease in lean tissue mass in some patients with cancer^26,27^. The essential role of adipose tissue wasting in the development of cancer cachexia is also evidenced by the fact that depletion of key enzymes in lipolysis alleviates cachectic phenotypes in mouse models^28^.

**Extended Data Fig. 10:**
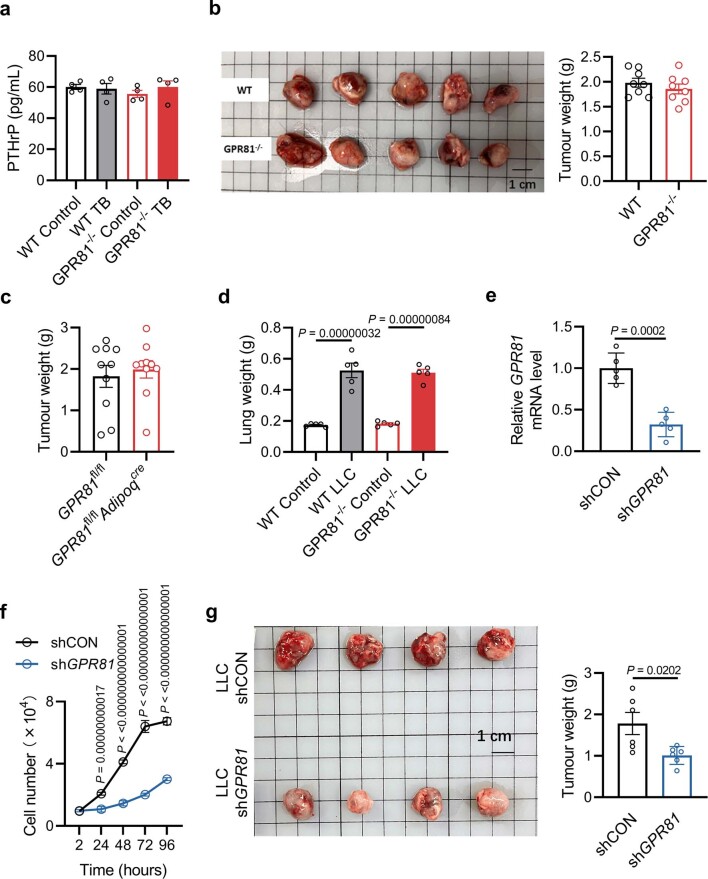
GPR81 ablation in host has no effect on tumour burden, whereas its deficiency in cancer cells retards tumour growth. **a**, Serum PTHrP levels of wild type (WT) and GPR81 knockout (GPR81^-/-^) mice with or without LLC xenograft tumours (n = 4 for each group). **b**, Representative image and averaged weight of tumours derived from WT and GPR81^-/-^ mice (n = 8 for each group). **c**, Averaged weight of tumours derived from *GPR81*^fl/fl^ and *GPR81*^fl/fl^*Adipoq*^*cre*^ mice (n = 10 for each group). **d**, Lung weight of WT tumour-free (WT Control), WT with LLC lung tumour (WT LLC), GPR81^-/-^ Control, and GPR81^-/-^ LLC mice in the orthotopic model (n = 5 for each group). **e**, Relative *GPR81* mRNA level in the LLC cells expressing GPR81-specific shRNA (sh*GPR81*) or control shRNA (shCON) (n = 5 biologically independent samples in each group). **f**, Cell number over 96 h showing the proliferation of the LLC cells expressing shCON or sh*GPR81* (n = 5 biologically independent samples in each group). **g**, Representative image and averaged weight of tumours derived from LLC cells expressing shCON or sh*GPR81* (n = 6 for each group). All data are presented as the mean ± s.e.m. *P* values were determined by one-way ANOVA with Tukey’s multiple comparisons (a, d), two-tailed unpaired Student’s *t*-test (b, c, e, g), or two-way ANOVA (f). Source data

Mechanistically, we have provided multiple lines of evidence to define a lactate-stimulated cachectic pathway, which sequentially activates the GPR81–Gαi/o-Gβγ–RhoA/ROCK1–p38 signalling cascade, rather than the well-established positive regulator of thermogenesis and lipolysis, namely PKA^29,30^. A variety of physiological stimuli, including cold^19,31^ and starvation^32^, converge on PKA to stimulate lipid mobilization. In addition, previous studies have shown that parathyroid hormone-related peptide (PTHrP)^7^ and lipid-mobilizing factor/plasma protein zinc-α2-glycoprotein^33^ induce adipose wasting via activating the PKA-dependent pathway. It is worth mentioning that we did not observe the upregulation of PTHrP in our study (Extended Data Fig. 10a), which is consistent with previous reports showing no increases in PTHrP levels in animal models or patients with cachexia^34,35^. Paradoxically, GPR81 is reported as a Gαi/o-coupled receptor that mediates the anti-lipolytic effect of insulin via suppressing adenylyl cyclase–cAMP–PKA activation in response to acute lactate stimulation^12,13^. If that were the case, activation of GPR81 by lactate would be expected to inhibit rather than activate adipose wasting due to inhibition of PKA. However, our data explicitly indicate that chronic elevation of blood lactate level is sufficient to trigger WAT browning and lipolysis, as well as muscle atrophy and body weight loss. Consistently, it has been reported that prolonged lactate infusion (> 3 h) in healthy humans leads to increased thermogenesis, EE and plasma free fatty acid and glycerol levels^36^. Moreover, PKA inhibitor does not block the reduction of body weight or WAT browning induced by lactate infusion, indicating that PKA does not contribute to the lactate-induced cachexia. In contrast, using phosphoproteomics and pharmacological analysis, we have demonstrated that lactate sequentially activates the GPR81–RhoA/ROCK1–p38 cascade that conveys the signalling of lactate/GPR81 to promote WAT browning. This is based on the facts that: (1) the activity levels of p38 and ROCK1 are repressed in the iWAT of the GPR81^−/−^ tumour-bearing mice; (2) lactate-induced p38 activation and subsequent UCP1 upregulation are profoundly suppressed by inhibition of RhoA/ROCK1; and (3) ablation of UCP1 ameliorates body weight loss and adipose and muscle remodelling induced by LLC tumours. It is also noteworthy that the phosphoproteomics data unveil enhanced activation of ERK1/2 in the GPR81^−/−^ iWAT. Unlike p38 that promotes adipose tissue browning and thermogenesis in response to various stresses^37^, ERK1/2 is mainly activated by growth stimuli and involved in the regulation of adipogenesis^14^. Because diminished adipogenesis has been observed in cancer cachexia^38^, the increased activation of ERK1/2 in GPR81-deficient mice may contribute to the sustained adipogenesis, thus alleviating tumour-induced and lactate-induced adipose wasting.

The current study has marked the host GPR81 as the major cancer cachexia-causing factor. In addition to being a catabolic driver, emerging evidence has shown that stimulation of GPR81 with lactate plays a role in tumour growth. For instance, inhibiting GPR81 expression represses the proliferation of breast and pancreatic cancer cells^39,40^. GPR81 is also implicated in regulating the tumour microenvironment via autocrine and paracrine functions^41–43^. However, if GPR81 expression in LLC cells was intact, the tumours were similar in size whether derived from WT or GPR81^−/−^ mice, or from *GPR81*^fl/fl^ or *GPR81*^fl/fl^*Adipoq*^*cre*^ mice (Extended Data Fig. 10b,c). Tumour growth in the orthotopic model was also comparable in the WT and GPR81^−/−^ mice, as indicated by the similar lung weights in the mice with cancer (Extended Data Fig. 10d). These results strongly indicate that the palliation of cachectic symptoms in GPR81^−/−^ mice is mainly attributed to GPR81 deficiency in the host. Nevertheless, knocking down GPR81 expression in LLC cells substantially repressed cancer cell proliferation in vitro and tumour growth in vivo (Extended Data Fig. 10e–g). Therefore, lactate/GPR81 propels the progression of both cancer and its complication of cachexia and worsens the prognoses of patients, while blocking GPR81 may have dual therapeutic benefits in treating both cancer and cancer cachexia.

In summary, this work reveals chronic activation of GPR81 by lactate instigates adipose metabolic remodelling manifested as enhanced browning and lipolysis via the Gαi/o-Gβγ–RhoA/ROCK1–p38 signalling cascade, which promotes muscle dystrophy and systemic hypercatabolism. Thus, targeting GPR81 and its key signal components holds great promise to combat metabolic impairments in cancer cachexia for the improvement of life quality and lifespan of patients with cachexia.

## Methods

### Human serum samples

Blood samples were collected from lung adenocarcinoma patients at Dazhou Central Hospital in Sichuan, China. This study was approved by the ethics committee of the Dazhou Central Hospital, and written informed consent was obtained from all patients before sample collection. The detailed information of patients is provided in Supplementary Tables 1 and 2. The patient IDs in these tables were arbitrary identifiers assigned by the researchers, which did not represent the IDs in medical records. Blood samples were centrifuged at 1,000*g* for 10 min. Then serum was transferred to a new tube and stored in liquid nitrogen before analysis. The serum lactate level was determined using Biosen C-Line glucose lactate analyser (EKF Diagnostics, 5213-0051-6200).

### Mice

Mice were maintained on a 12-h light–dark cycle (07:00–19:00 light on) at room temperature of 23 ± 2 °C with 40–60% humidity, and food (Xietong Shengwu, 1010083) and water were provided ad libitum. The animal facility is certified by the Association for Assessment and Accreditation of Laboratory Animal Care. All procedures involving animals conformed to the Guide for the Care and Use of Laboratory Animals (National Institutes of Health publication no. 86-23, revised 2011) and were approved by the Institutional Animal Care and Use Committee of Peking University (protocol no. IMM-XiaoRP-13). *Kras*^*LSL-G12D/+*^;*p53*^*R172H/+*^ mice (strain name: C57BL/6JSmoc-Trp53em4(R172H) Krasem4(LSL-G12D)) were purchased from Shanghai Model Organisms Center. GPR81^−/−^ mice were generated as previously described^44^ and backcrossed to the C57BL/6J background for at least eight times. GPR81^fl/fl^ mice were purchased from GemPharmatech (stock no. T006370), and crossed with Adipoq-cre mice (Jackson Laboratories, stock no. 028020) to generate *GPR81*^fl/fl^ *Adipoq*^*cre*^ with adipose-specific knockout of GPR81. The UCP1^−/−^ mice were purchased from Jackson Laboratories (stock no. 003124). GPR81^fl/fl^, Adipoq-cre and UCP1^−/−^ were all on the C57BL/6J background.

The cachexia model was generated according to the previous reports^34,45–47^. Male mice aged 8 to 10 weeks old were randomized into a control tumour-free or a tumour-bearing group. In the tumour-bearing group, 1 × 10^6^ LLC cells in 100 μl PBS were subcutaneously injected into the right flank of mice. The tumour-free group received 100 μl PBS. In the other xenograft mouse models of B16-F10, EMT6 and MIA PaCa-2, 1 × 10^6^ cells were subcutaneously injected into C57BL/6J, BALB/c and BALB/c nude mice, respectively. All animals were monitored carefully throughout the study. Mice were euthanized if the tumour size reached 1,500 mm^3^ or ulcerated. For the orthotopic model of LLC, 1 × 10^6^ LLC cells were injected into C57BL/6J mice via a tail vein. For the genetically engineered mouse model, 10-week-old *Kras*^*LSL-G12D/+*^;*p53*^*R172H/+*^ male mice were randomly assigned to two groups, receiving 2.5 × 10^7^ plaque-forming units of adenovirus expressing Cre recombinase (HanBio, HH20230208GX-AP01) or containing empty expression vector via intranasal injection once^5,47^. Mice were euthanized 8 weeks after adenovirus administration.

Rectal temperature was measured using a rectal temperature probe (Physitemp, BAT-12). Body composition was determined by Body Composition Analyzer (EchoMRI, 500).

### Metabolic characterization

Mice were single-housed in metabolic cages (Columbus Instruments, CLAMS) for 24 h to adapt to the environment before measurement. EE was recorded for 24 h of one light–dark cycle. Analysis of covariance was used to analyse the differences in EE with body weight treated as an independent variable. The corrected EE was assessed based on regression-based analysis and analysis of covariance^48,49^. Food intake was recorded over the entire 48 h.

### Delivery of lactate with osmotic minipumps

Osmotic minipumps (Alzet, 2001) loaded with PBS, 1,600 mg ml^−1^ or 800 mg ml^−1^ sodium l-lactate (Sigma-Aldrich, 71718) or 1,600 mg ml^−1^ sodium d-lactate (Sigma-Aldrich, 71716) were implanted subcutaneously on the back when mice were under anaesthesia. For the 14-day infusion, the minipumps loaded with PBS or 1,600 mg ml^−1^ sodium l-lactate were removed after 7 days and replaced with a new one and infused for another week. Body weights were recorded, and the blood lactate level was determined with Lactate Scout Analyzer (EKF Diagnostics, Lactate Scout 4) and lactate test strips (Kirgen, XC-rssz72).

### Grip strength

Limb strength was assessed using a digital force gauge (Chatillon, model DFX II). Mice were positioned with forelimb paws grabbing a bar attached to a force transducer and were pulled back horizontally by the tail away from the bar until they released their grip. The test for each mouse was repeated for five times with a 120 s pause between each measurement. The grip strength was determined by averaging the readings from five repetitions.

### Treatment with inhibitors

For H89 treatment, the injection of H89 (10 mg per kg body weight in 100 μl PBS; MedChemExpress, HY-15979A) was started the day after implantation of the minipump and was given once every 2 days for 6 days, during which the body weight was recorded. Ten days after subcutaneous xenograft of LLC cells, p38 inhibitor SB203580 (2.5 mM; MedChemExpress, 152121-47-6), ROCK1 inhibitor Y-27632 (2.5 mM; Solarbio, IY0050) or Gβγ inhibitor gallein (2.5 mM; MedChemExpress, HY-D0254) were injected into the inguinal fat pads of mice. All the inhibitors were dissolved in PBS and injected once every 2 days, and PBS was used as a vehicle control. Body weights of mice were recorded during the treatment. At the ending point, mice were euthanized, and tissues were isolated for further analysis.

### [U-^13^C] sodium lactate intravenous infusion and sample preparation

Uniformly labelled ^13^C [U-^13^C] sodium lactate (20% wt/wt, CLM-1579-0.5, CIL) was diluted in PBS to a final concentration of 5% (wt/wt). Mice were fasted from 8:00 to 14:00 and infused with 5% [U-^13^C] sodium lactate through a tail vein at 0.1 μl g^−1^ min^−1^ for 2 h. Mice were then euthanized immediately, and blood and tissue samples were collected^50,51^. Blood samples were centrifuged at 1,000*g* for 10 min at 4 °C to collect serum. To extract metabolites from serum samples, 80 μl 80% methanol (Merck, 1.06007.4008) was added into 20 μl serum, and the mixture was centrifugated at 15,000*g* for 10 min. To extract metabolites from tissue, tissue samples were quickly dissected and grounded in 80% methanol followed by centrifugation at 15,000*g* for 10 min. The supernatant was kept at −80 °C until liquid chromatography–mass spectrometry (LC–MS) analysis.

### Histology, immunofluorescence and immunohistochemistry

Tissue samples of iWAT, gWAT, BAT and skeletal muscle were rapidly fixed in 4% paraformaldehyde (PFA) in PBS. The PFA-fixed tissues were dehydrated, embedded in paraffin and cut into 5 μm sections. H&E staining was performed according to the standard procedure. Adipose tissue sections were incubated in the citric acid antigen repair buffer (10 mM sodium citrate, 0.05% Tween 20, pH 6.0) and blocked with 1% BSA (Sigma-Aldrich, A9056) for 30 min. Then, the sections were incubated with primary antibody against UCP1 (1:50 dilution; Cell Signaling, 72298S) at 4 °C overnight, washed three times with TBST, followed by incubation with goat anti-rabbit Alexa Fluor 594 (1:200 dilution Invitrogen, A-11012) for 1 h at room temperature. For immunofluorescence of adipose tissue from patients, primary antibodies against phospho-p38 MAPK (1:200 dilution; Cell Signaling, 4511T) and p38 MAPK (1:200 dilution; Affinity, BF8015) were used. Goat anti-rabbit Alexa Fluor 568 antibody (1:200 dilution; Invitrogen, A-11011) and goat anti-mouse Alexa Fluor 647 antibody (1:200 dilution; Invitrogen, A-21235) were used as secondary antibodies. The stained slides were imaged under a fluorescence microscope (Olympus, BX-53). For immunohistochemistry of adipose tissue, the tissue sections were incubated with primary antibody against UCP1 (1:500 dilution; Sigma-Aldrich, U6382) and horseradish peroxidase-conjugated Goat anti-Rabbit IgG (H + L; 1:500 dilution; Invitrogen, 31460) was used as secondary antibody. The signal intensity of all the images was analysed using ImageJ software.

### Interstitial fluid collection and lactate detection

Tissue interstitial fluid was collected from fresh inguinal adipose tissue. Around 0.1 g adipose tissue was minced in 500 μl PBS and filtered through a 0.22-μm nylon filter^52^. The fluid was collected, and lactate level was determined using Biosen C-Line glucose lactate analyser.

### Measurement of NEFAs, glycerol and cytokines

The levels of NEFAs in the serum were measured using an automatic biochemical analyser (Roche, cobas c311). Levels of glycerol were determined by a Glycerol Assay Kit (Nanjing Jiancheng Bioengineering Institute, F005-1-1). Levels of TNF-α, IFN-γ, IL-1β and IL-6 in adipose tissue lysate were assessed by ELISA (eBioscience, ProcartaPlex multiple cytokines ELISA kit, PPX-06).

### Cell lines

LLC1 (CRL-1642), B16-F10 (CRL-6475), EMT6 (CRL-2755) and MIA PaCa-2 (CRL-1420) were purchased from the American Type Culture Collection. Cells were cultured in DMEM (Solarbio, 11965) supplemented with 10% FBS (Gibco, 10099141C) and penicillin–streptomycin (Gibco, 15070063). Cells were maintained in a 37 °C incubator under 5% CO_2_. To silence the expression of GPR81 in LLC cells, LLC cells were infected with lentivirus expressing GFP as well as nonspecific shRNA (shCon: TTCTCCGAACGTGTCACGTAA) or shRNA targeting *GPR81* (shGPR81: CCTGGAAGTCAAGCACTAT). Polybrene (4 μg ml^−1^, Solarbio, H8761) was used to increase efficiency. LLC cell clones were first selected with puromycin (5 μg ml^−1^; Pharmabiology, P32076) and then isolated using flow cytometry. GPR81 knockdown efficiency was confirmed by RT–qPCR.

### Isolation of primary preadipocytes

Mouse SVF of WAT was isolated from 14-day-old C57BL/6J mice. Inguinal adipose tissue was minced and digested in type 2 collagenase (2 mg ml^−1^) for 1 h at 37 °C. The digestion was ended by adding an equal volume of DMEM/F12 (Gibco, C11330500BT) supplied with 10% FBS to the digestion mixture. The suspension was centrifuged at 110*g* for 5 min and the supernatant was removed. Cells were resuspended and filtered through a 75-μm cell strainer, and then plated onto a culture dish and maintained in DMEM/F12 supplied with 10% FBS.

### Differentiation of SVF into adipocytes

When 100% confluency was reached, SVF cells were induced with the adipogenic cocktail containing 1 μM dexamethasone (Sigma-Aldrich, D4902), 10 μg ml^−1^ insulin (Sigma, I9278) and 0.5 mM isobutylmethylxanthine (Sigma, I7018) supplemented in DMEM/F12 for 2 days, followed by incubation in the adipocyte culture medium (DMEM supplied with 10% FBS) containing 10 μg ml^−1^ insulin for another 2 days. Then the cells were maintained in DMEM supplied with 10% FBS for 2 days. For treatment with inhibitors, cells were fasted for 12 h and pretreated with gallein (10 μM; MedChemExpress, HY-D0254), Y-27632 (10 μM; Solarbio, IY0050), SB203580 (10 μM; MedChemExpress, 152121-47-6), PTX (100 ng ml^−1^; List Biological Laboratories, 180) or H89 (20 μM; MedChemExpress, HY-15979A) for 4, 1, 1, 12 or 1 h, respectively, before treatment with 20 mM sodium l-lactate.

### siRNA transfection

siRNAs were designed by GenePharma (Shanghai, China) and transfected into SVF-derived adipocytes using Lipofectamine RNAiMAX (Thermo Fisher Scientific, 13778030). Nonspecific siRNA was used as a control. Sequences of siRNAs are listed in Supplementary Table 3.

### Immunofluorescence staining of cultured adipocytes

SVF-derived adipocytes were treated with 20 mM sodium l-lactate or PBS for 30 min before fixing in 4% PFA for 15 min at room temperature. After washed three times with PBS, cells were permeabilized with 0.1% Triton X-100 (Sigma-Aldrich, T9284-100ML) in PBS for 10 min, and blocked in 5% BSA for 30 min at room temperature. The primary antibodies against RhoA (1:200 dilution; Santa Cruz, sc-418) and rhotekin (1:200 dilution; Affinity, DF9868) were applied at 4 °C overnight. Cells were rinsed in PBS and incubated with secondary antibodies for 1 h at room temperature. DAPI (1:500 dilution; Solarbio, S2110) was used to stain the nuclei. After staining, cells were imaged using a confocal microscope (Zeiss, LSM880), and signal intensity was quantified using ImageJ.

### Immunoblotting

For tissue lysate preparation, about 100 mg tissue sample was weighed and homogenized in 500 μl RIPA lysis buffer (20 mM HEPES, 150 mM sodium chloride, 2 mM EDTA, 1% Triton X-100, 0.1% SDS, 10% glycerol, 0.5% sodium deoxycholate; Solarbio, R0010) supplemented with protease and phosphatase inhibitors (Solarbio, P1261). The tissue lysates were incubated at 4 °C for 30 min and centrifuged at 15,000*g* for 10 min. For cell protein extraction, 1 × 10^6^ cells were lysed in 500 μl RIPA lysis buffer and sonicated by a sonic oscillator (Vibracell, CV00188) before centrifugation at 15,000*g* for 10 min. Protein concentration was determined by BCA Protein Assay Kit (Thermo Scientific, 23227). The protein extract was resolved by SDS–PAGE, then transferred onto a PVDF membrane (Bio-Rad, 1620177). Membranes were blocked with 5% BSA for 1 h at room temperature and incubated with the indicated specific antibodies overnight at 4 °C. After secondary antibody incubation, chemiluminescent signals were detected by a gel imaging system (Tanon, 5200). Signal intensity was quantified using ImageJ. All the antibodies were purchased from Cell Signaling Technology, including the antibodies against phospho-p38 MAPK (1:1,000 dilution; 4511T), p38 MAPK (1:1,000 dilution; 8690T), ATF2 (1:2,000 dilution; 35031), phospho-PKA substrate (1:1,000 dilution; 9624S) and β-tubulin (1:1,000 dilution; 2146S), except for the antibodies against phospho-ATF2 (1:2,000 dilution; Abcam, ab32019), UCP1 (1:5,000 dilution; Abcam, ab209483) and GPR81 (1:1,000 dilution; Novus, NBP1-51956). Goat anti-Rabbit IgG (H + L; 1:10,000 dilution; Invitrogen, 31460) or Rabbit anti-Goat IgG (H + L; 1:10,000 dilution; Invitrogen, 31402) was used as secondary antibody. Protein levels were normalized to β-tubulin expression.

### RT–qPCR

Total RNA was isolated from tissue samples or cells using TRIzol Lysis Reagent (Invitrogen, 15596026). A total of 2 μg of total RNA was used to synthesize cDNA using a kit (Transgen, AE311) according to the manufacturer’s instruction. Primer sequences for genes of interest are listed in the Supplementary Table 4. Relative quantification of mRNA level was determined by the 2^−(ΔΔCt)^ method using the Prism 7500 SDS software (Thermo Fisher Scientific) in LightCycler 96 (Roche), and 18S rRNA was used as the internal reference.

### RNA-seq and bioinformatics analysis

Total RNA was extracted from adipose tissue and quadriceps muscle for library preparation. The samples were then sequenced by Illumina NovaSeq 6000 (PE150, from Berry Genomics). After pre-mapping sequencing quality evaluation by FastQC (version: 0.10.1), the reads were mapped to the mouse reference genome (genome version: mm10) with hisat2 (version: 2.1.0) to get uniquely mapped reads using default parameters. Htseq-count from HTSeq (version: 0.9.1) was used to calculate gene count matrix, and StringTie (version: 1.3.3b) was used to calculate gene expression values (fragments per kilobase per million mapped fragments, FPKM). Differential gene expression was analysed using DEseq2 tool (version: 1.18.1). The genes with low expression across all samples (FPKM < 0.5 in all samples) were removed, and the genes with adjusted *P* value below 0.05 were extracted as significantly differentially expressed genes.

### Untargeted metabolomics

Around 100 μl human serum sample was mixed with 400 μl methanol (Merck, 1.06007.4008) by vortexing and centrifugation at 4 °C and 15,000*g*, for 10 min. Then, 200 μl of supernatant was dried by SpeedVac (Thermo Fisher Scientific, SPD1010) and stored at −80 °C. The dried sample was redissolved in 50 μl ultrapure water and analysed using UPLC–MS immediately. Untargeted LC–MS profiling of polar metabolites was performed on a UPLC platform (Waters Corp, ACQUITY I-Class) coupled with tandem ESI-QTOF mass spectrometry (Waters Corp, Synapt G2-Si). In total, 2 μl of each sample was separated on a BEH Amide column (1.7 µm, 2.1 × 150 mm i.d. Waters Corp) at 45 °C. The mobile phase A was 0.1% formic acid in H_2_O, and B was 0.1% formic acid in acetonitrile. The elution gradient started at 99% B from 0 to 0.1 min, increased to 70% B from 0.2 to 7.0 min, and maintained at 99% B from 7.1 to 10 min. The flow rate was 0.4 ml min^−1^. MSe scan was performed in negative mode with a mass range of 50 to 1,200 *m/z* at a resolution of 10,000. The capillary voltages and cone voltages were set as 2.0 kV and 20 V, respectively. The source temperature was 120 °C, and the desolvation temperature was 500 °C. The desolvation gas flow was 800 l h^−1^. The acquired MS data were generated using the Progenesis QI software (Waters Corp) under a standard protocol. Each ion was announced by retention time and the *m/z* data pairs (RT–*m/z*) as an observed compound peak, and the intensity of the peak was integrated. The online Human Metabolome Database (HMDB; https://www.hmdb.ca/) was used to align the molecular mass data (*m/z*) and label the possible metabolite. The peak of lactate was confirmed by lactate standard (100 µM in H_2_O) under identical LC–MS conditions of untargeted metabolic analysis. The representative LC–MS spectrum of lactate standard is shown in the Extended Data Fig. 1c,d. In addition, we used the LC-QQQ (88.9 > 42.8) to quantitatively compare subsequent samples. The concentrations of the lactate in samples were calculated using a calibration curve generated from the standard solution (Extended Data Fig. 1e).

### Phosphoproteomics

Phosphoproteomics was performed as previously reported^53^. Briefly, 200 μg protein was extracted from adipose tissue and digested by trypsin (Hualishi Scientific, China). Peptides were desalted with tC18 Cartridges (Sep-Pak, Waters), dried by the SpeedVac, and stored at −80 °C.

To enrich phosphopeptides, the desalted peptides were resuspended in 200 μl binding buffer (1 M lactic acid, 50% acetonitrile, 1% trifluoroacetic acid) and incubated with titanium dioxide beads (GL Sciences). The phosphopeptides eluted from the titanium dioxide beads were then dried using a SpeedVac.

Phosphopeptides were analysed by the Easy-nLC 1200 system coupled to an Orbitrap Fusion Lumos LC–MS/MS system (Thermo Fisher Scientific). Raw data were submitted for database search by SEQUEST HT built-in Proteome Discoverer (Thermo Fisher Scientific, version 2.2) using Uniprot mouse protein database (version 2021-02-18, 21990 sequences). Up to two missed cleavages were allowed for trypsin digestion. Carbamidomethylation of cysteine, N-terminal acetylation of protein, oxidation of methionine and phosphorylation of serine/threonine/tyrosine were set as variable modifications. Maximum peptide and protein false discovery rates were both limited to 1%.

The probability of location of phosphosite was determined by the ptmRS module within Proteome Discoverer and phosphopeptides harbouring phosphosites with a probability of more than 75% were quantified with area of chromatographic peaks. A phosphosite with the ratio of means of normalized quantitative values from the comparing groups >1.2 or <1/1.2 and the adjusted *P* value < 0.05 were considered as differentially expressed phosphosites. The kinase activities were estimated by the kinase–substrate enrichment analysis algorithm. Kinase–substrate relationships was obtained from PhosphoSite, Phospho.ELM, SIGNOR 2.0 (ref. ^54^) and NetworKIN^55^. The information of substrate motifs was obtained from either the literature^56^ or Motif-X^57^.

### Statistical analysis

The collection of experimental data followed a randomized approach. The ‘RAND’ function of Excel was used to get a random value for each animal and ranked the animals in the order of that random value. Then each experimental group was assigned one animal from the queue in order until all the animals were allocated. No statistical methods were used to predetermine sample sizes, but our sample sizes are similar to those reported in previous publications^58,59^. The diameters of individual adipocytes were measured using ImageJ. Results were analysed with GraphPad Prism 9.0.1. Data distribution was assumed to be normal, but this was not formally tested. The statistical comparisons between two groups were determined by unpaired two-tailed Student’s *t*-test or one-way ANOVA, and two-way ANOVA was used for multiple comparisons. *P* values below 0.05 were considered significant. The survival rate difference was analysed by simple survival analysis (Kaplan–Meier). Correlation between blood/serum lactate level and body weight change was examined using Pearson simple linear regression.

### Reporting summary

Further information on research design is available in the Nature Portfolio Reporting Summary linked to this article.

## Supplementary information

Supplementary Information Supplementary Fig. 1 and Tables 1–4.

Reporting Summary

Supplementary Data Source data for Supplementary Fig. 1.

## Source data

Source Data Fig. 1 Statistical source data.

Source Data Fig. 2 Statistical source data.

Source Data Fig. 2 Unprocessed western blots.

Source Data Fig. 3 Statistical source data.

Source Data Fig. 4 Statistical source data.

Source Data Fig. 4 Unprocessed western blots.

Source Data Fig. 5 Statistical source data.

Source Data Fig. 5 Unprocessed western blots.

Source Data Fig. 6 Statistical source data.

Source Data Fig. 6 Unprocessed western blots.

Source Data Extended Data Fig. 1 Statistical source data.

Source Data Extended Data Fig. 2 Statistical source data.

Source Data Extended Data Fig. 3 Statistical source data.

Source Data Extended Data Fig. 4 Statistical source data.

Source Data Extended Data Fig. 5 Statistical source data.

Source Data Extended Data Fig. 5 Unprocessed western blots.

Source Data Extended Data Fig. 6 Statistical source data.

Source Data Extended Data Fig. 6 Unprocessed western blots.

Source Data Extended Data Fig. 7 Statistical source data.

Source Data Extended Data Fig. 8 Statistical source data.

Source Data Extended Data Fig. 8 Unprocessed western blots.

Source Data Extended Data Fig. 10 Statistical source data.

## Data Availability

RNA-seq data are deposited in Genome Sequence Archive under accession number CRA009143. Phosphoproteomic and untargeted metabolomics data are deposited in the Archive for Miscellaneous Data (OMIX). The accession number of phosphoproteomic data is OMIX002525. The accession number of untargeted metabolomics of murine sera is OMIX002520. The accession number of untargeted metabolomics of sera from patients with lung cancer is OMIX002511. The databases used in the study are HMDB (https://www.hmdb.ca/), Uniprot mouse protein database (https://www.uniprot.org/taxonomy/10090), PhosphoSite (https://www.phosphosite.org/psrSearchAction), Phospho.ELM (http://phospho.elm.eu.org), SIGNOR 2.0 (http://signor.uniroma2.it/) and NetworKIN (http://networkin.info/). Source data are provided with this paper.
