# Author Correction: Activation of GPR81 by lactate drives tumour-induced cachexia

**DOI:** 10.1038/s42255-024-01207-4

**Published:** 2024-12-14

**Authors:** Xidan Liu, Shijin Li, Qionghua Cui, Bujing Guo, Wanqiu Ding, Jie Liu, Li Quan, Xiaochuan Li, Peng Xie, Li Jin, Ye Sheng, Wenxin Chen, Kai Wang, Fanxin Zeng, Yifu Qiu, Changlu Liu, Yan Zhang, Fengxiang Lv, Xinli Hu, Rui-Ping Xiao

**Affiliations:** 1 Institute of Molecular Medicine, College of Future Technology, Peking University, Beijing, China; 2 Dazhou Central Hospital, Sichuan, China; 3 Peking-Tsinghua Center for Life Sciences, Peking University, Beijing, China; 4 Sanford Burnham Prebys Medical Discovery Institute, La Jolla, CA USA; 5 State Key Laboratory of Membrane Biology, Peking University, Beijing, China; 6 Beijing City Key Laboratory of Cardiometabolic Molecular Medicine, Peking University, Beijing, China; 7 PKU-Nanjing Institute of Translational Medicine, Nanjing, China

**Keywords:** Cancer, Molecular medicine, Homeostasis, Cancer metabolism

Correction to: *Nature Metabolism* 10.1038/s42255-024-01011-0, published online 18 March 2024.

In the version of the article initially published, in Extended Data Fig. 2a, the line colours for the control and LLC groups were reversed and have now been amended in the HTML and PDF versions of the article.

