# Supplementary material for: Activation of GPR81 by lactate drives tumour-induced cachexia
Source: Nat Metab. 2024 Mar 18;6(4):708–23. doi: 10.1038/s42255-024-01011-0 (PMC11052724; doi:10.1038/s42255-024-01011-0)
Supplement: Supplementary file 1 — Supplementary Fig. 1 and Tables 1–4. [file 42255_2024_1011_MOESM1_ESM.pdf]

nature metabolism

Article https://doi.org/10.1038/s42255-024-01011-0

Activation of GPR81 by lactate drives tumour-induced cachexia

In the format provided by the authors and unedited

Supplementary Table

Supplementary Table 1. Clinical information of lung adenocarcinoma patients in the study of correction between blood lactate level and body weight change.

Patient ID	Stage
P2-C2	IV
P3-N1	I
P4-N2	I
P5-C3	IV
P6-C4	IV
P7-N3	I
P8-C5	IV
BJ02	IV
BJ03	IV
BJ04	IV
BJ05	IV
BJ07	IV
BJ09	IV
BJ11	III
BJ14	IV
BJ18	IV
BJ21	IV
BJ22	III
BJ23	IV
BJ24	IV
DZ201	IV
DZ202	IV
DZ203	IV
DZ204	IIIB
DZ205	I
DZ206	IB

Supplementary Table 2. Clinical information of lung adenocarcinoma patients in the study of blood lactate level change after surgical removal of lung tumor.

Patient ID	Stage
01	I
11	II
14	I
38	I
47	I
51	II
53	I
55	I
58	I
59	II
60	I
69	I
76	I
77	I
81	I
91	I
96	I
97	I
99	I
100	I
105	I
112	I
121	I
124	I
126	II
129	I
132	I
135	II
136	I
142	I
149	I
159	I
163	I
164	I
166	I
168	I

Supplementary Table 3. List of siRNAs sequences.

Target Gene	Forward primer 5' – 3'	Reverse primer 5' – 3'
siControl	UUCUCCGAACGUGUCACGUTT	ACGUGACACGUUCGGAGAATT
p38 MAPK	CGCCAGAGAUCAUGCUAAATT	UUUAGCAUGAUCUCUGGCGTT
RhoA	CUUAUCCAGACACCGAUGUTT	ACAUCGGUGUCUGGAUAAGTT
ROCK1	GCGUUUGCCAAUAGUCCUUTT	AAGGACUAUUGGCAAACGCTT
Gnb1	GGGCAUCUGGCAAAGAUUUTT	AAAUCUUUGCCAGAUGCCCTT
Gnb2	CCCAGGACGGAAAGCUCAUTT	AUGAGCUUUCCGUCCUGGGTT
GPR132	GGCUACCACUACCUGCGUUTT	AACGCAGGUAGUGGUAGCCTT
GPR4	GCAACAUCUAUAUCAGCAUTT	AUGCUGAUAUAGAUGUUGCTT
GPR81	CCUGGAAGUCAAGCACUAUTT	AUAGUGCUUGACUUCCAGGTT
MCT1	CCAAAUCCAUCACUGUCUUTT	AAGACAGUGAUGGAUUUGGTT
MCT4	GGUCUUUGUGGUGAGCUAUTT	AUAGCUCACCACAAAGACCTT

Supplementary Table 4. List of primer sequences for qPCR..

Gene	Forward primer 5' – 3'	Reverse primer 5' – 3'
18s	GGAAGGGCACCACCAGGAGT	TGCAGCCCCGGACATCTAAG
GPR81	GAATGCCATCTCCAACCGGA	GGCTCCAAACAACGTTGACC
UCP1	CAAAAACAGAAGGATTGCCGAAA	TCTTGGACTGAGTCGTAGAGG
Dio2	TCAGGTAACAATTATGCCTCGGA	GCTGAACCAAAGTTGACCACC
HSL	GATTTACGCACGATGACACAGT	ACCTGCAAAGACATTAGACAGC
Cidea	TGACATTCATGGGATTGCAGAC	CATGGTTTGAAACTCGAAAAGGG
Acox1	TCCAGACTTCCAACATGAGGA	CTGGGCGTAGGTGCCAATTA
Acsl1	CGCACCCTTCCAACCAACA	CGCTATTTCCACTGACTGCAT

Supplementary Figures

Supplementary Fig 1. Phosphoproteomics analysis of iWAT from tumor-bearing mice.

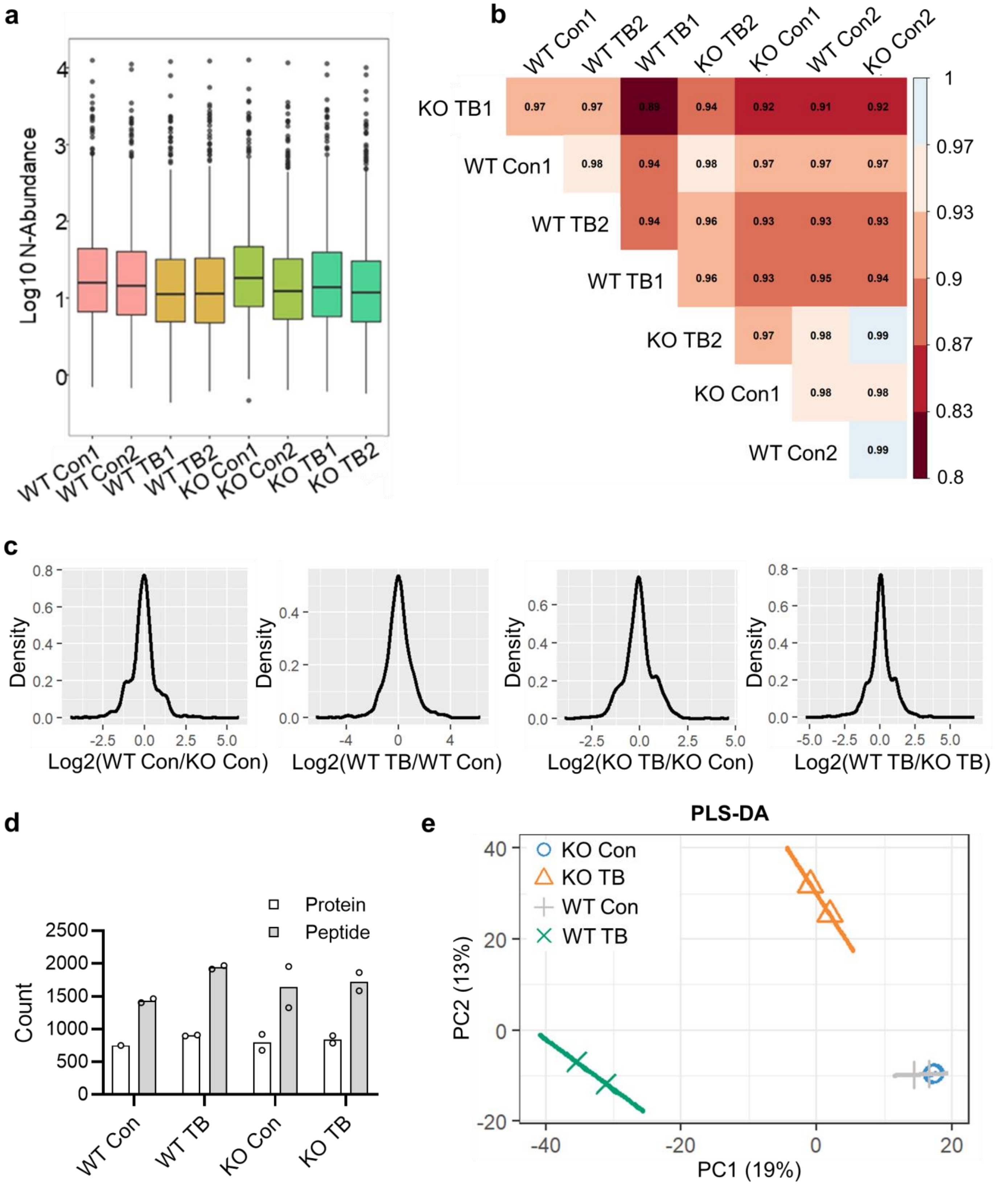

a, Box plots showing the relative abundance of quantified phosphosites in all samples. The bounds of box is defined by IQR (Interquartile Range), range from quartile 1 (Q1) to Q3. The centre of box is Q3. The whiskers is 1.5 times of IQR. The exact value was shown in the Supplementary Data 1. (WT, wild type; KO, GPR81$^{-/-}$; Con, tumor-free; TB, LLC tumor-bearing, n=2 biologically independent samples).

b, Quantitative Pearson correlation analysis of phosphosites among samples.

c, Density plots of the fold change of phosphosites abundance by comparing WT Con with KO Con, WT TB with WT Con, KO TB with KO Con and WT TB with KO TB.

d, Counts of identified phosphorylated proteins and peptides in all samples.

e, PLS-DA showing the clustering of inguinal white adipose tissue (iWAT) samples from WT and GPR81$^{-/-}$ mice with or without tumors.
