# Supplementary material for: Activation of GPR81 by lactate drives tumour-induced cachexia
Source: Nat Metab. 2024 Mar 18;6(4):708–23. doi: 10.1038/s42255-024-01011-0 (PMC11052724; doi:10.1038/s42255-024-01011-0)
Supplement: Supplementary file 2 — Reporting Summary [file 42255_2024_1011_MOESM2_ESM.pdf]

nature portfolio

Corresponding author(s): Xinli Hu, Rui-Ping Xiao

Last updated by author(s): Jan 26, 2024

Reporting Summary

Nature Portfolio wishes to improve the reproducibility of the work that we publish. This form provides structure for consistency and transparency in reporting. For further information on Nature Portfolio policies, see our Editorial Policies and the Editorial Policy Checklist.

Statistics

For all statistical analyses, confirm that the following items are present in the figure legend, table legend, main text, or Methods section.

n/a	Confirmed	
☐	☒	The exact sample size (n) for each experimental group/condition, given as a discrete number and unit of measurement
☐	☒	A statement on whether measurements were taken from distinct samples or whether the same sample was measured repeatedly
☐	☒	The statistical test(s) used AND whether they are one- or two-sided<br>*Only common tests should be described solely by name; describe more complex techniques in the Methods section.*
☐	☒	A description of all covariates tested
☐	☒	A description of any assumptions or corrections, such as tests of normality and adjustment for multiple comparisons
☐	☒	A full description of the statistical parameters including central tendency (e.g. means) or other basic estimates (e.g. regression coefficient) AND variation (e.g. standard deviation) or associated estimates of uncertainty (e.g. confidence intervals)
☐	☒	For null hypothesis testing, the test statistic (e.g. F, t, r) with confidence intervals, effect sizes, degrees of freedom and P value noted<br>*Give P values as exact values whenever suitable.*
☒	☐	For Bayesian analysis, information on the choice of priors and Markov chain Monte Carlo settings
☒	☐	For hierarchical and complex designs, identification of the appropriate level for tests and full reporting of outcomes
☒	☐	Estimates of effect sizes (e.g. Cohen's d, Pearson's r), indicating how they were calculated

Our web collection on statistics for biologists contains articles on many of the points above.

Software and code

Policy information about availability of computer code

Data collection	Real-Time PCR: LightCycler96 Real-Time PCR systems (Roche).<br>Chemiluminescent signals detection: Tanon 5200 Chemiluminescent Imaging System.<br>Untargeted metabolomics: UPLC system (Waters Corp, ACQUITY I-Class) coupled with tandem ESI-QTOF mass spectrometry (Waters Corp, Synapt G2-Si).<br>Phosphoproteomics: Easy-NLC 1200 liquid chromatography system (Thermo Fisher Scientific, USA) coupled to Orbitrap Fusion Lumos LC-MS/MS system (Thermo Fisher Scientific, USA).<br>RNA-seq:Illumina NovaSeq 6000 (PE150, from Berry Genomics).<br>Metabolic characterization: CLAMS Metabolic cages (Columbus Instruments).<br>Body Composition Analyzer (EchoMRI, 500), BioTek Multi-Detection Microplate Reader, Olympus BX51 Microscopy, ZEISS LSM 880 Microscopy.
Data analysis	GraphPad Prism 9.0.1, Microsoft Excel 2021, ImageJ 1.53q.<br>RNA-Seq: FastQC (v0.10.1), hisat2(v2.1.0), HTSeq (v0.9.1) , StringTie (v1.3.3b), DESeq2 (v1.18.1).<br>Mouse reference genome (genome version: mm10)<br>The untargeted metabolomics data pretreatment including peak deconvolution, retention time correction, fragment extraction, isotopes and adducts annotation: Progenesis QI software (Waters Corp. USA).<br>The online HMDB database (https://www.hmdb.ca).<br>Raw mass spectrometry data of phosphoproteomics was searched against the Uniprot mouse protein database (version 2021-02-18, 21990 sequences, https://www.uniprot.org/taxonomy/10090) with SEQUEST HT search engine built in Proteome Discoverer (PD) (Thermo Fisher Scientific, version 2.2)<br>The information of kinase-substrate relationships was obtained from publicly available databases: PhosphoSite (https://

www.phosphosite.org/psrSearchAction), Phospho.ELM(http://phospho.elm.eu.org), SIGNOR 2.0 (http://signor.uniroma2.it/), and NetworKIN(http://networkin.info).
Analysis of KSEA dataset: Motif-X (version:1.2)

For manuscripts utilizing custom algorithms or software that are central to the research but not yet described in published literature, software must be made available to editors and reviewers. We strongly encourage code deposition in a community repository (e.g. GitHub). See the Nature Portfolio guidelines for submitting code & software for further information.

Data

Policy information about availability of data

All manuscripts must include a data availability statement. This statement should provide the following information, where applicable:

- Accession codes, unique identifiers, or web links for publicly available datasets
- A description of any restrictions on data availability
- For clinical datasets or third party data, please ensure that the statement adheres to our policy

The source data of the Figures and Extended Data Figures are provided as a Source data files. RNA-seq data is deposited in Genome Sequence Archive (GSA) and accession number is CRA009143. Phosphoproteomic and untargeted metabolomics data are deposited in Archive for Miscellaneous Data (OMIX). The accession number of phosphoproteomic data is OMIX002525. The accession number of untargeted metabolomics of sera from mouse is OMIX002520. The accession number of untargeted metabolomics of sera from patients with lung cancer is OMIX002511.

Human research participants

Policy information about studies involving human research participants and Sex and Gender in Research.

Reporting on sex and gender	In the study of correction between lactate and body weight loss, the blood samples of 26 lung adenocarcinoma patients were collected, including 5 female and 21 male. In the study of lactate change after the surgical removal of lung tumor, the blood samples of 36 lung adenocarcinoma patients were collected,including 13 female and 23 male. Since the difference in prevelence of cancer cachexia in male or female have not been reported yet, we did not consider sex or gender in sample collection or analysis.
Population characteristics	In the study of correction between lactate and body weight loss, the human blood samples of 26 lung adenocarcinoma patients were collected. Mean body weight was 60.86±9.71kg. In the study of lactate change after the surgical removal of lung tumor, samples from 36 lung adenocarcinoma patients were collected. The detailed information about patients was listed in Supplementary Table 1, 2.
Recruitment	The human blood samples were collected from Dazhou Central Hospital in Sichuan province. We collected blood samples from as many lung adenocarcinoma paitents as we could. The patients suffered from metabolic or other diseases that may affect appetite were excluded. Metabolic disorders, such as diabetes, may cause changes in the levels of metabolites in serum. Other conditions, such as depression, may affect patient's appetite and lead to changes in body weight.
Ethics oversight	This study was approved by Ethics Committee of the Dazhou Central Hospital. Written informed consent was obtained from all patients before the blood collection.

Note that full information on the approval of the study protocol must also be provided in the manuscript.

Field-specific reporting

Please select the one below that is the best fit for your research. If you are not sure, read the appropriate sections before making your selection.

☒ Life sciences ☐ Behavioural & social sciences ☐ Ecological, evolutionary & environmental sciences

For a reference copy of the document with all sections, see nature.com/documents/nr-reporting-summary-flat.pdf

Life sciences study design

All studies must disclose on these points even when the disclosure is negative.

Sample size	Sample sizes were determined based on previous studies (doi: 10.1038/s41467-017-00726-x; doi: 10.1016/j.cmet.2014.06.011; doi:10.1038/nature13528) and to ensure the power of statistical analysis and minimize the use of experimental animals. The sample sizes of experiments were indicated in legends.
Data exclusions	No data were excluded.
Replication	Every experiment was repeated independently at least three times. All attempts at replication were successful.
Randomization	Mice were randomly allocated into groups for cancer cells injection, minipump implantation, or drug treatment.<br>As for lung adenocarcinoma patients, those with body weigh loss in the past 6 months were assigned to cachexia group and those without weight loss were assigned into non-cachexia group.

In the in vitro experiments, cells were plated in to culture dishes under the same c condition before treatment. The cells in different dish/well were allocated to different treatment group randomly.

Blinding

Blinding was not done in most of the studies since the data collection and analysis were performed by researchers knowing the design of the experiments.

Reporting for specific materials, systems and methods

We require information from authors about some types of materials, experimental systems and methods used in many studies. Here, indicate whether each material, system or method listed is relevant to your study. If you are not sure if a list item applies to your research, read the appropriate section before selecting a response.

Materials & experimental systems

n/a	Involved in the study	
☐	☒	Antibodies
☐	☒	Eukaryotic cell lines
☒	☐	Palaeontology and archaeology
☐	☒	Animals and other organisms
☒	☐	Clinical data
☒	☐	Dual use research of concern

Methods

n/a	Involved in the study	
☒	☐	ChIP-seq
☒	☐	Flow cytometry
☒	☐	MRI-based neuroimaging

Antibodies

Antibodies used

Antibodies for immunoblot:
Rabbit anti-UCP1 (Abcam, Cat. #ab209483) 1:5000
Rabbit anti-phospho-ATF2 (Abcam, Cat. #ab32019) 1:2000
Rabbit anti-phospho-p38 MAPK (Cell Signaling, Cat. #4511T) 1:1000
Rabbit anti-p38 MAPK (Cell Signaling, Cat. #8690T) 1:1000
Rabbit anti-ATF2 (Cell Signaling, Cat. #35031) 1:2000
Rabbit anti-Phospho-PKA Substrate (Cell Signaling, Cat. #9624S)1:1000
Rabbit anti-β-tubulin (Cell Signaling, Cat. #2146s) 1:1000
Goat anti-GPR81 (Novus, Cat. #NBP1-51956) 1:1000
Goat anti-Rabbit IgG (H+L) Secondary Antibody (Invitrogen, Cat. #31460) 1:10000
Rabbit anti-Goat IgG (H+L) Secondary Antibody (Invitrogen, Cat. #31402) 1:10000
Antibodies for immunofluorescence:
Rabbit anti-UCP1 (Cell Signaling, Cat. #72298S) 1:50
Mouse anti-p38 MAPK (Affinity, Cat. #BF8015) 1:200
Rabbit anti-phospho-p38 MAPK (Cell Signaling, Cat. #4511T) 1:200
Alexa Fluor 594 goat anti-rabbit lgG(H+L) (Invitrogen, Cat. #A-11012) 1:200
Alexa Fluor 568 goat anti-rabbit lgG(H+L) (Invitrogen, Cat. #A-11011) 1:200
Alexa Fluor 647 goat anti-mouse lgG(H+L) (Invitrogen, Cat. #A-21235) 1:200
Mouse anti-Rho A (26C4) (Santa Cruz Biotech., Cat. #sc-418) 1:200
Rabbit anti-RTKN (Affinity, Cat. #DF9868) 1:200
Antibodies for immunohistochemistry:
Rabbit anti-UCP1 (Sigma-Aldrich, Cat. #U6382) 1:500
Goat anti-Rabbit IgG (H+L) Secondary Antibody (Invitrogen, Cat. #31460) 1:500

Validation

All antibodies were purchased from commercial campanies. The antibodies have been tested by the manufacturers.
Antibodies for immunoblot:
Rabbit anti-UCP1 (Abcam, Cat. #ab209483) can be found in 26 citations. The manufacturer provides antibody testing data: https://www.abcam.cn/ucp1-antibody-epr20381-ab209483/reviews/82865
Rabbit anti-phospho-ATF2 (Abcam, Cat. #ab32019) can be found in 9 citations. The manufacturer provides antibody testing data: https://www.abcam.cn/products/primary-antibodies/atf2-phospho-t71-antibody-e268-ab32019.html
Rabbit anti-phospho-p38 MAPK (Cell Signaling, Cat. #4511T) can be found in 3149 citations. The manufacturer provides antibody testing data: https://www.cellsignal.cn/products/primary-antibodies/phospho-p38-mapk-thr180-tyr182-d3f9-xp-rabbit-mab/4511
Rabbit anti-p38 MAPK (Cell Signaling, Cat. #8690T) can be found in 1957 citations. The manufacturer provides antibody testing data: https://www.cellsignal.cn/products/primary-antibodies/p38-mapk-d13e1-xp-rabbit-mab/8690
Rabbit anti-ATF2 (Cell Signaling, Cat. #35031) can be found in 22 citations. The manufacturer provides antibody testing data: https://www.cellsignal.cn/products/primary-antibodies/atf-2-d4l2x-xp-rabbit-mab/35031
Rabbit anti-Phospho-PKA Substrate (Cell Signaling, Cat. #9624S)can be found in 343 citations. The manufacturer provides antibody testing data: https://www.cellsignal.cn/products/primary-antibodies/phospho-pka-substrate-rrxs-t-100g7e-rabbit-mab/9624
Rabbit anti-β-tubulin (Cell Signaling, Cat. #2146s) can be found in 943 citations. The manufacturer provides antibody testing data: https://www.cellsignal.cn/products/primary-antibodies/b-tubulin-antibody/2146
Goat anti-GPR81 (Novus, Cat. #NBP1-51956)can be found in 2 citations. The manufacturer provides antibody testing data: https://www.novusbio.com/products/gpr81-antibody_nbp1-51956
Rabbit anti-Goat IgG (H+L) Secondary Antibody (Invitrogen, Cat. #31402) can be found in 186 citations. The manufacturer provides antibody testing data: https://www.thermofisher.cn/cn/zh/antibody/product/Rabbit-anti-Goat-IgG-H-L-Secondary-Antibody-Polyclonal/31402
Goat anti-Rabbit IgG (H+L) Secondary Antibody (Invitrogen, Cat. #31460) can be found in 2521 citations. The manufacturer provides

antibody testing data: https://www.thermofisher.cn/cn/zh/antibody/product/Goat-anti-Rabbit-IgG-H-L-Secondary-Antibody-Polyclonal/31460
Rabbit anti-UCP1 (Cell Signaling Technology, Cat. #72298S) can be found in 11 citations. The manufacturer provides antibody testing data: https://www.cellsignal.cn/products/primary-antibodies/ucp1-e9z2v-xp-rabbit-mab/72298
Mouse anti-p38 MAPK (Affinity, Cat. #BF8015) can be found in 6 citations. The manufacturer provides antibody testing data: https://www.affbiotech.cn/goods-18184-BF8015-p38_MAPK_Monoclonal_Antibody.html
Alexa Fluor 594 goat anti-rabbit lgG(H+L) (Invitrogen, Cat. #A-11012) can be found in 2701 citations. The manufacturer provides antibody testing data: https://www.thermofisher.cn/cn/zh/antibody/product/Goat-anti-Rabbit-IgG-H-L-Cross-Adsorbed-Secondary-Antibody-Polyclonal/A-11012
Alexa Fluor 568 goat anti-rabbit lgG(H+L) (Invitrogen, Cat. #A-11011) can be found in 2697 citations. The manufacturer provides antibody testing data: https://www.thermofisher.cn/cn/zh/antibody/product/Goat-anti-Rabbit-IgG-H-L-Cross-Adsorbed-Secondary-Antibody-Polyclonal/A-11011
Alexa Fluor 647 goat anti-mouse lgG(H+L) (Invitrogen, Cat. #A-21235) can be found in 1476 citations. The manufacturer provides antibody testing data: https://www.thermofisher.cn/cn/zh/antibody/product/Goat-anti-Mouse-IgG-H-L-Cross-Adsorbed-Secondary-Antibody-Polyclonal/A-21235
Mouse anti-Rho A (26C4) (Santa Cruz Biotech., Cat. #sc-418) can be found in 1280 citations. The manufacturer provides antibody testing data: https://www.scbt.com/p/rho-a-antibody-26c4?requestFrom=search
Rabbit anti-RTKN (Affinity, Cat. #DF9868): The manufacturer provides antibody testing data: https://www.affbiotech.cn/goods-13341-DF9868-RTKN_Antibody.html
Rabbit anti-UCP1 (Sigma-Aldrich, Cat. #U6382) can be found in 156 citations. The manufacturer provides antibody testing data: https://www.sigmaaldrich.cn/CN/zh/product/sigma/u6382

Eukaryotic cell lines

Policy information about cell lines and Sex and Gender in Research

Cell line source(s)	LLC (Cat. #CRL-1642), EMT6 (Cat. #CRL-2755), MIA PaCa-2 (Cat. #CRL-1420) and B16-F10 (Cat. #CRL-6475) were obtained from American Type Culture Collection (ATCC). Mouse stromal vascular fraction (SVF) of white adipose tissue was isolated from 14-day old wild type or GPR81-/- mice (doi: 10.1038/s41590-021-01023-y).
Authentication	Cell lines used were not authenticated.
Mycoplasma contamination	All cell lines were tested negative for mycoplasma contamination.
Commonly misidentified lines (See ICLAC register)	No commonly misidentified cell lines were used.

Animals and other research organisms

Policy information about studies involving animals; ARRIVE guidelines recommended for reporting animal research, and Sex and Gender in Research

Laboratory animals	C57BL/6 mice (Stock NO.219), BALB/c Nude mice (stock NO.401) and BALB/c mice (stock NO.211) were purchased from Charles River. KrasLSL-G12D/+;p53R172H/+ mice (Strain name: C57BL/6JSmoc-Trp53em4(R172H) Krasem4(LSL-G12D)) were purchased from Shanghai Model Organisms Center, Inc.. GPR81-/- mice were generated as previously described (doi:10.1038/s41467-017-00726-x) and backcrossed to C57BL/6J background for at least 8 times. GPR81fl/fl strain (Stock NO. T006370) was purchased from GemPharmatech (Nanjing, China), and crossed with Adipoq-cre mice (Jackson Laboratories, Stock NO. 028020) to generate GPR81fl/fl Adipoqcre with adipose-specific knockout of GPR81. The UCP1-/- mice were purchased from Jackson Laboratories (Jackson Laboratories, Stock NO. 003124). C57BL/6, GPR81-/-, GPR81fl/fl Adipoqcre, and UCP1-/- male mice at age of 8-10 weeks were used for experiments. BALB/c Nude male mice at age of 5-6 weeks were used for experiments. Male KrasLSL-G12D/+;p53R172H/+ mice at age of 10 weeks were used for experiments. Mice were maintained on a 12-h light–dark cycle (07:00-19:00 light on), at room temperature of 23 ± 2°C with 40–60% humidity. Food and water were provided ad libitum. The animal facility is certified by Association for Assessment and Accreditation of Laboratory Animal Care (AAALAC). All procedures involving animals were conformed to the Guide for the Care and Use of Laboratory Animals (NIH publication No. 86-23, revised 2011), and were approved by the Institutional Animal Care and Use Committee (IACUC) of Peking University (Protocol# IMM-XiaoRP-13).
Wild animals	This study did not involve any wild animals.
Reporting on sex	Only male mice were analyzed. The gender differences in the prevalence of cancer cachexia have not been reported. Almost all of the previous studies have used male mice to generate cachexia models (doi: 10.1038/s41467-017-00726-x; doi: 10.1016/j.cmet.2014.06.011; doi:10.1038/nature13528; doi: 10.1016/j.cell.2015.08.031).
Field-collected samples	No field collected samples were used.
Ethics oversight	All procedures involving animals were conformed to the Guide for the Care and Use of Laboratory Animals (NIH publication No. 86-23, revised 2011), and were approved by the Institutional Animal Care and Use Committee (IACUC) of Peking University (Protocol# IMM-XiaoRP-13).

Note that full information on the approval of the study protocol must also be provided in the manuscript.
