# Supplementary figures and images for: Activation of GPR81 by lactate drives tumour-induced cachexia
Source: Nat Metab. 2024 Mar 18;6(4):708–23. doi: 10.1038/s42255-024-01011-0 (PMC11052724; doi:10.1038/s42255-024-01011-0)

Raw data of western blots in Fig. 2h.

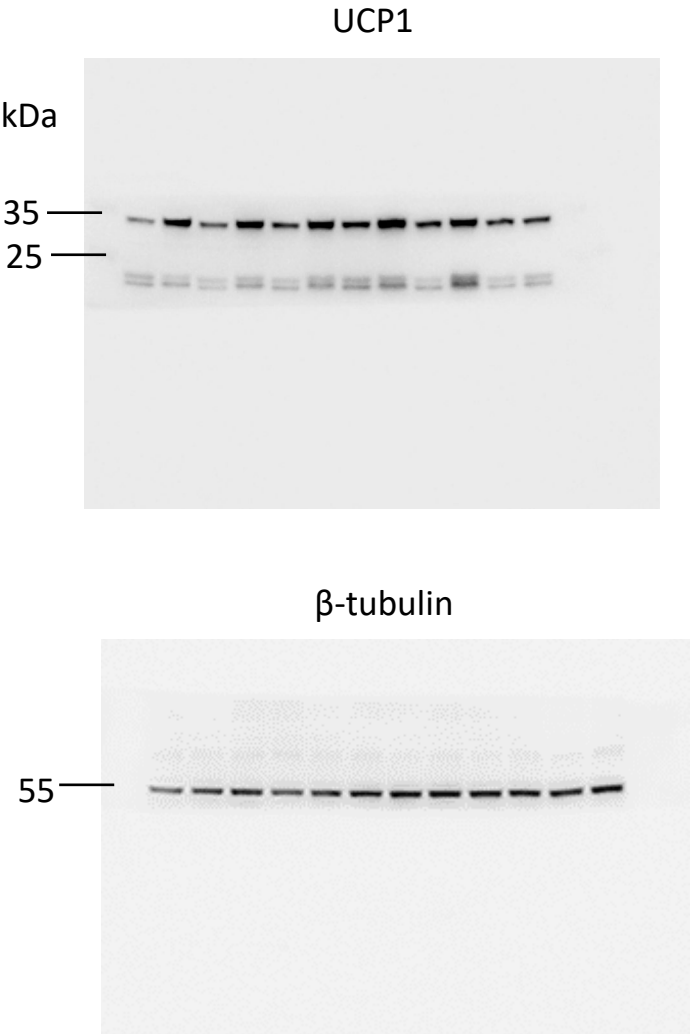

**Fig. 2h:** UCP1 and β-tubulin.

Supplement: Supplementary file 6 — Unprocessed western blots. [file 42255_2024_1011_MOESM6_ESM.pdf]

Raw data of western blots in Fig. 4d.

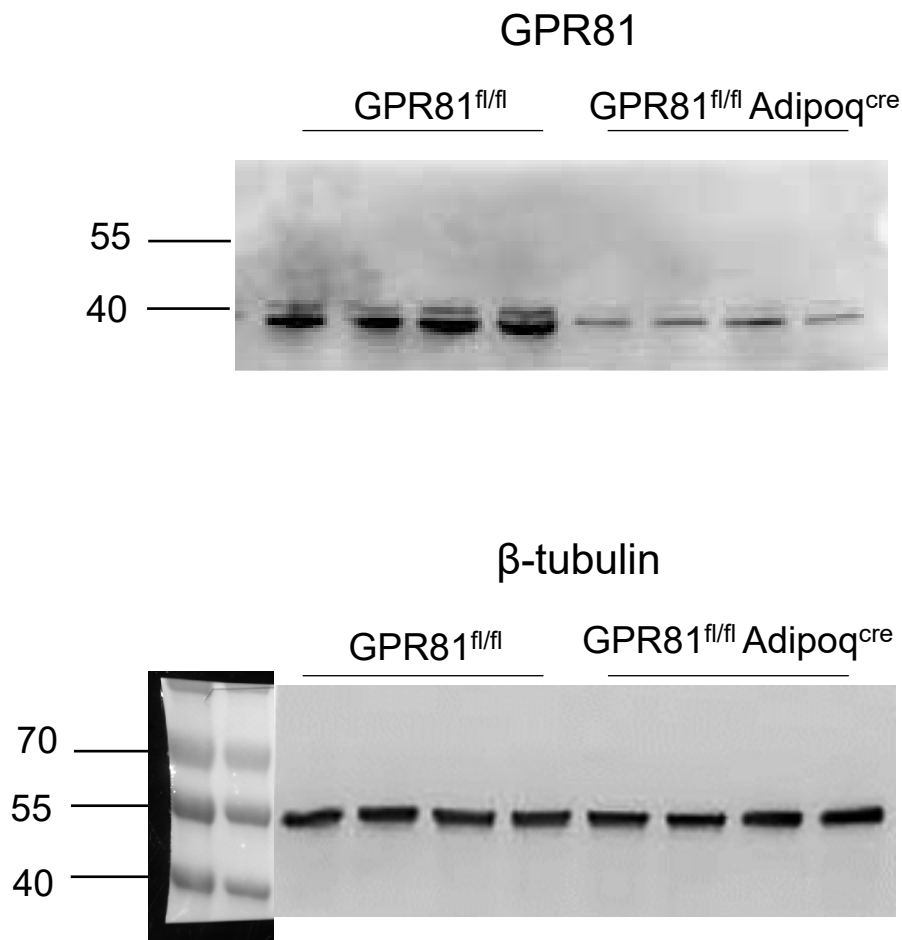

**Fig. 4d:** GPR81 and β-tubulin.

Supplement: Supplementary file 9 — Unprocessed western blots. [file 42255_2024_1011_MOESM9_ESM.pdf]

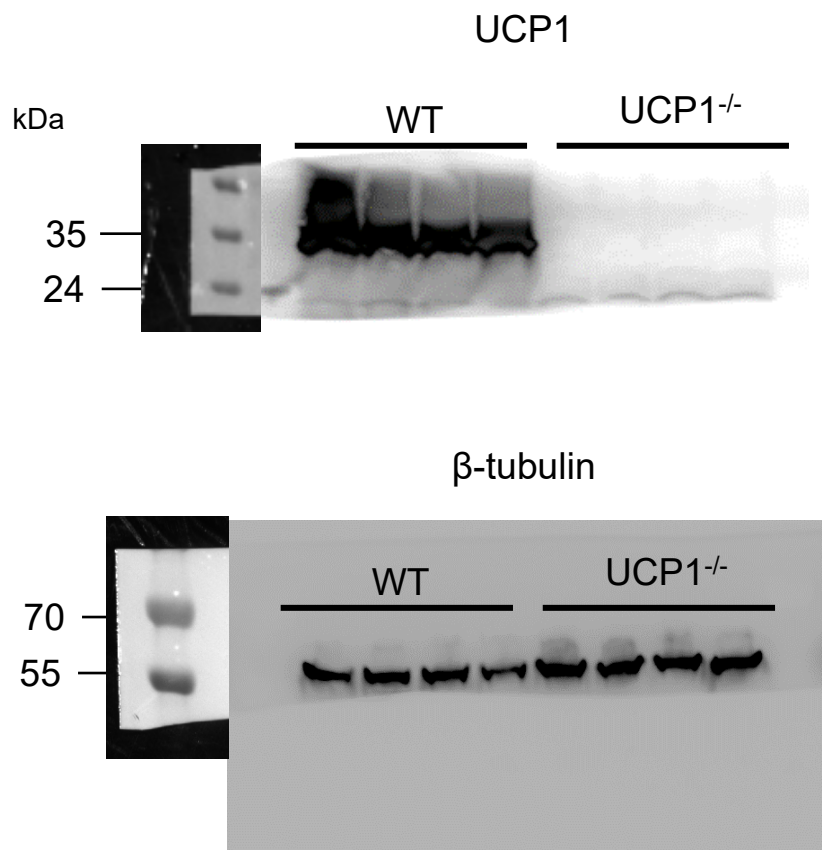

**Extended Data Fig. 5e:** UCP1 and  $\beta$ -tubulin.

Supplement: Supplementary file 19 — Unprocessed western blots. [file 42255_2024_1011_MOESM19_ESM.pdf]

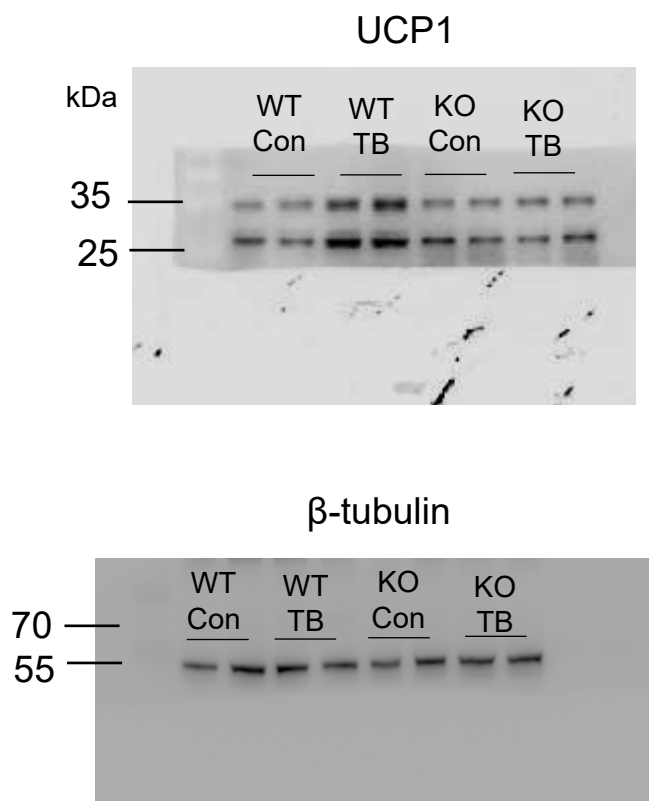

**Extended Data Fig. 6e:** UCP1 and  $\beta$ -tubulin.

Supplement: Supplementary file 21 — Unprocessed western blots. [file 42255_2024_1011_MOESM21_ESM.pdf]
