# Supplementary material for: Activation of GPR81 by lactate drives tumour-induced cachexia
Source: Nat Metab. 2024 Mar 18;6(4):708–23. doi: 10.1038/s42255-024-01011-0 (PMC11052724; doi:10.1038/s42255-024-01011-0)
Supplement: Supplementary file 11 — Unprocessed western blots. [file 42255_2024_1011_MOESM11_ESM.pdf]

Raw data of western blots in Fig. 5c.

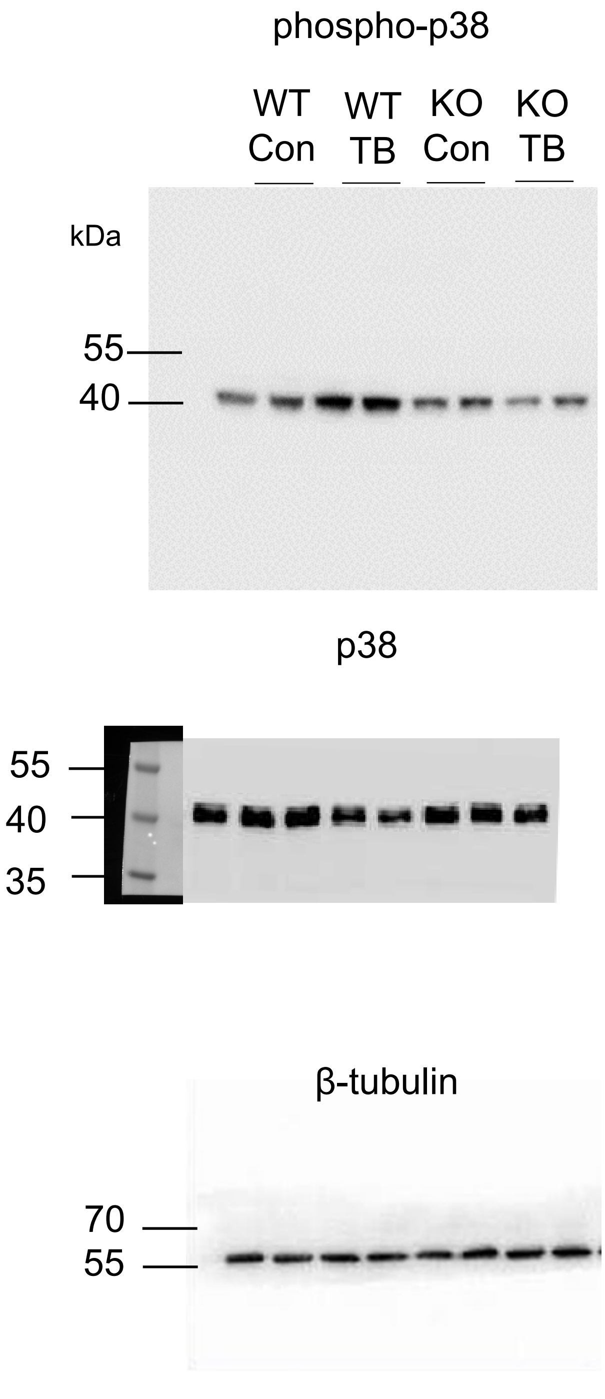

Fig. 5c: phospho-p38, p38 and β-tubulin.

Raw data of western blots in Fig. 5f.

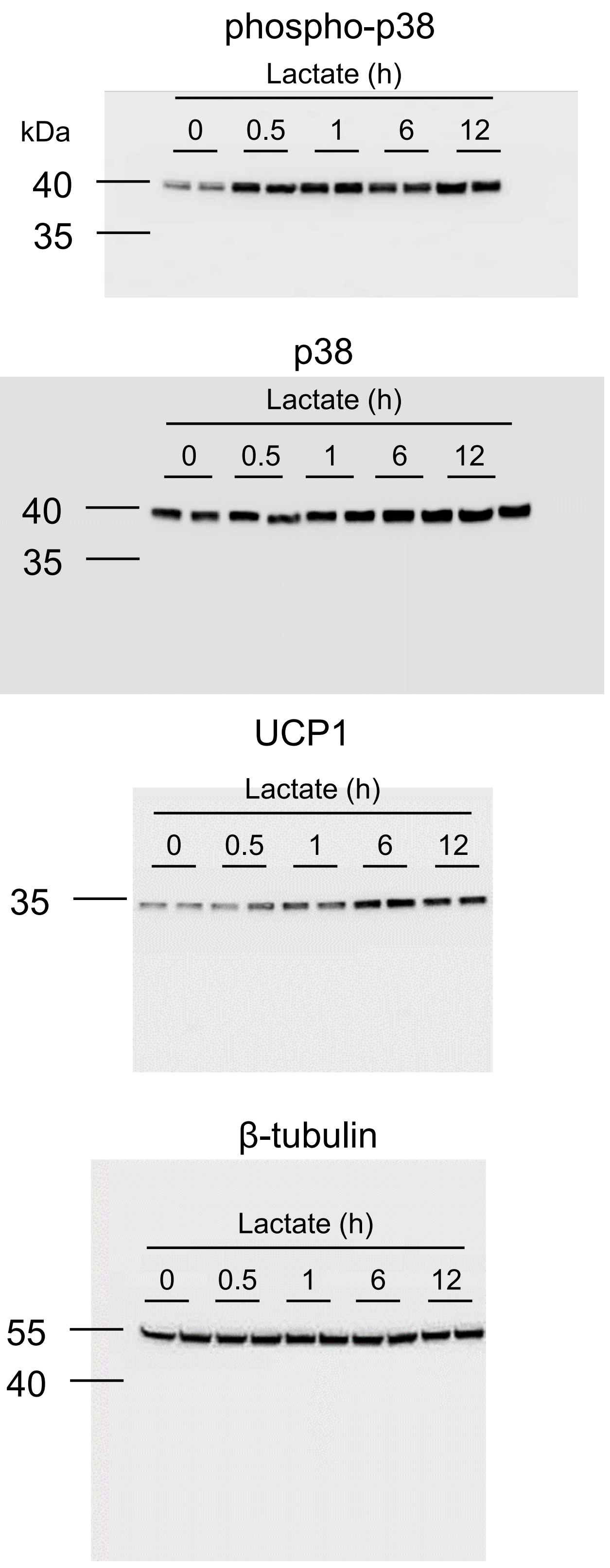

Fig. 5f: phospho-p38, p38, UCP1 and β-tubulin.

Raw data of western blots in Fig. 5g.

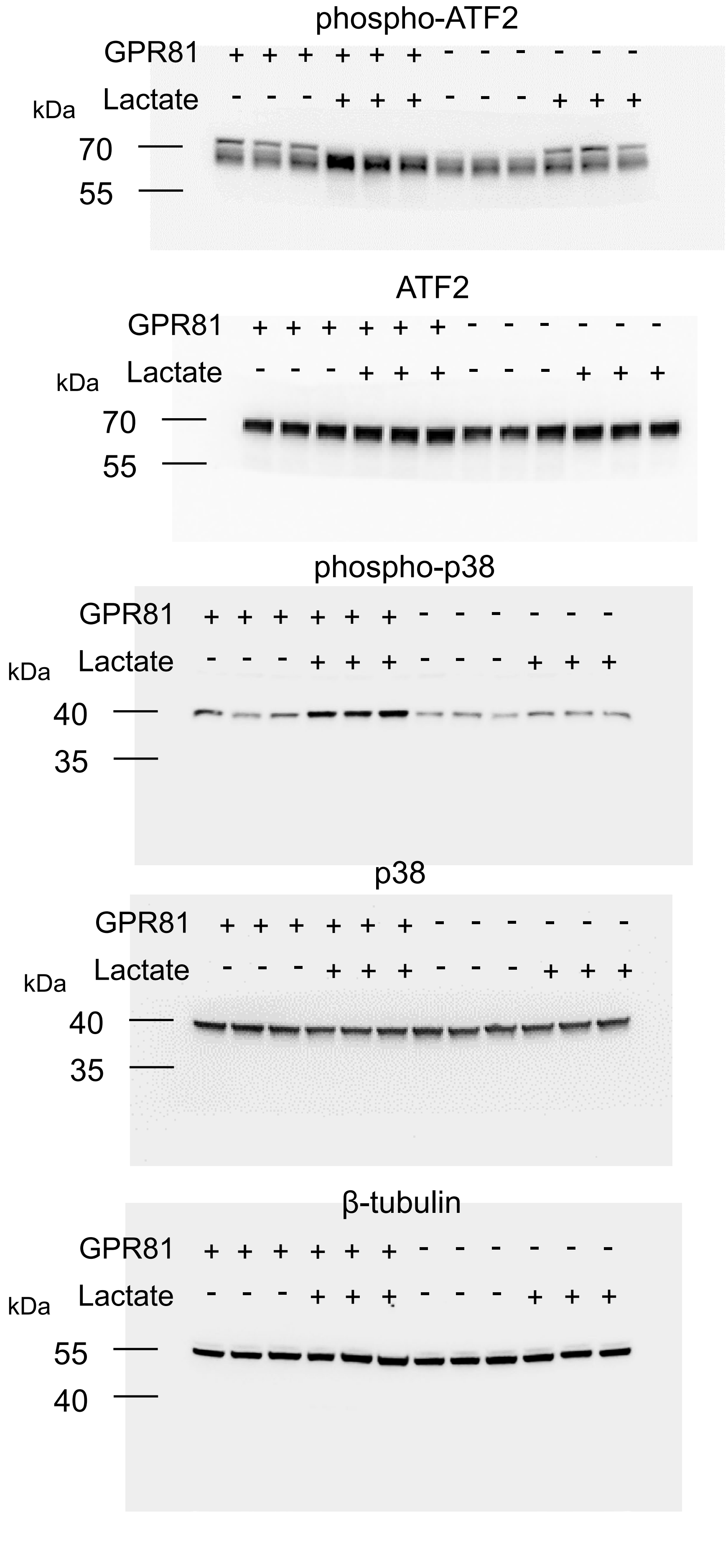

Fig. 5g: phospho-ATF2, ATF2, phospho-p38, p38 and β-tubulin.

Raw data of western blots in Fig. 5h.

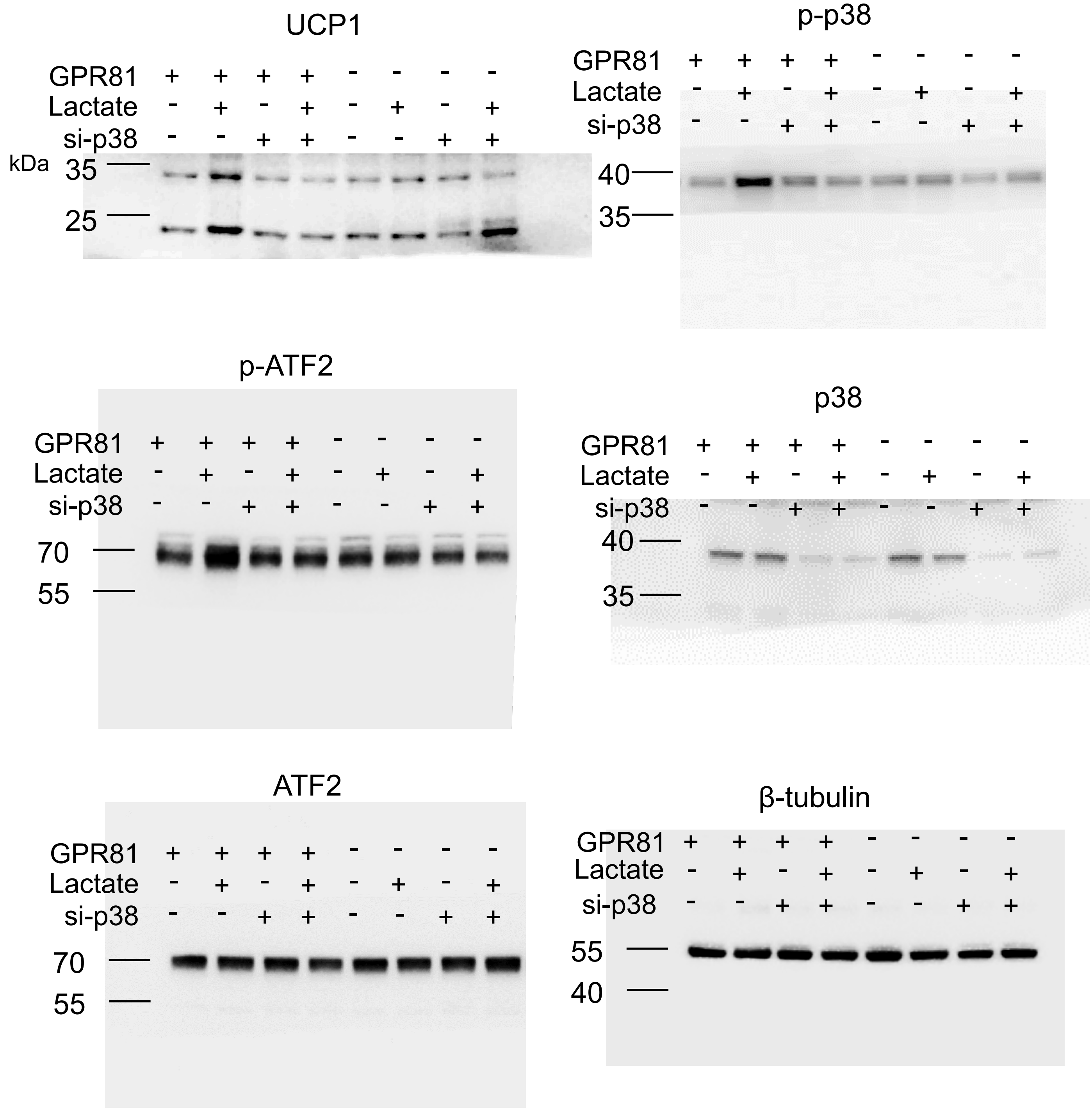

Fig. 5h: phospho-ATF2, ATF2, phospho-p38, p38, UCP1 and β-tubulin.
