# Supplementary material for: Activation of GPR81 by lactate drives tumour-induced cachexia
Source: Nat Metab. 2024 Mar 18;6(4):708–23. doi: 10.1038/s42255-024-01011-0 (PMC11052724; doi:10.1038/s42255-024-01011-0)
Supplement: Supplementary file 13 — Unprocessed western blots. [file 42255_2024_1011_MOESM13_ESM.pdf]

Raw data of western blots in Fig. 6a.

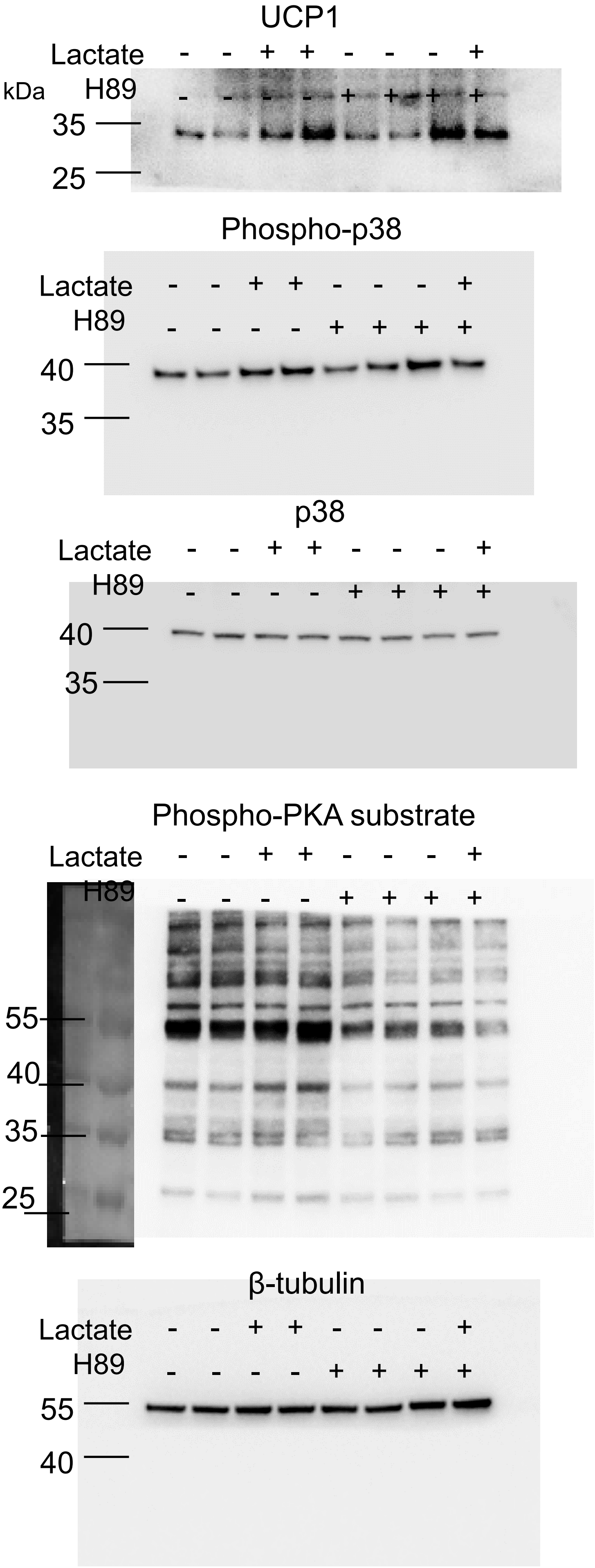

Fig. 6a: UCP1, phospho-ATF2, ATF2, phospho-p38, p38 and β-tubulin.

Raw data of western blots in Fig. 6c

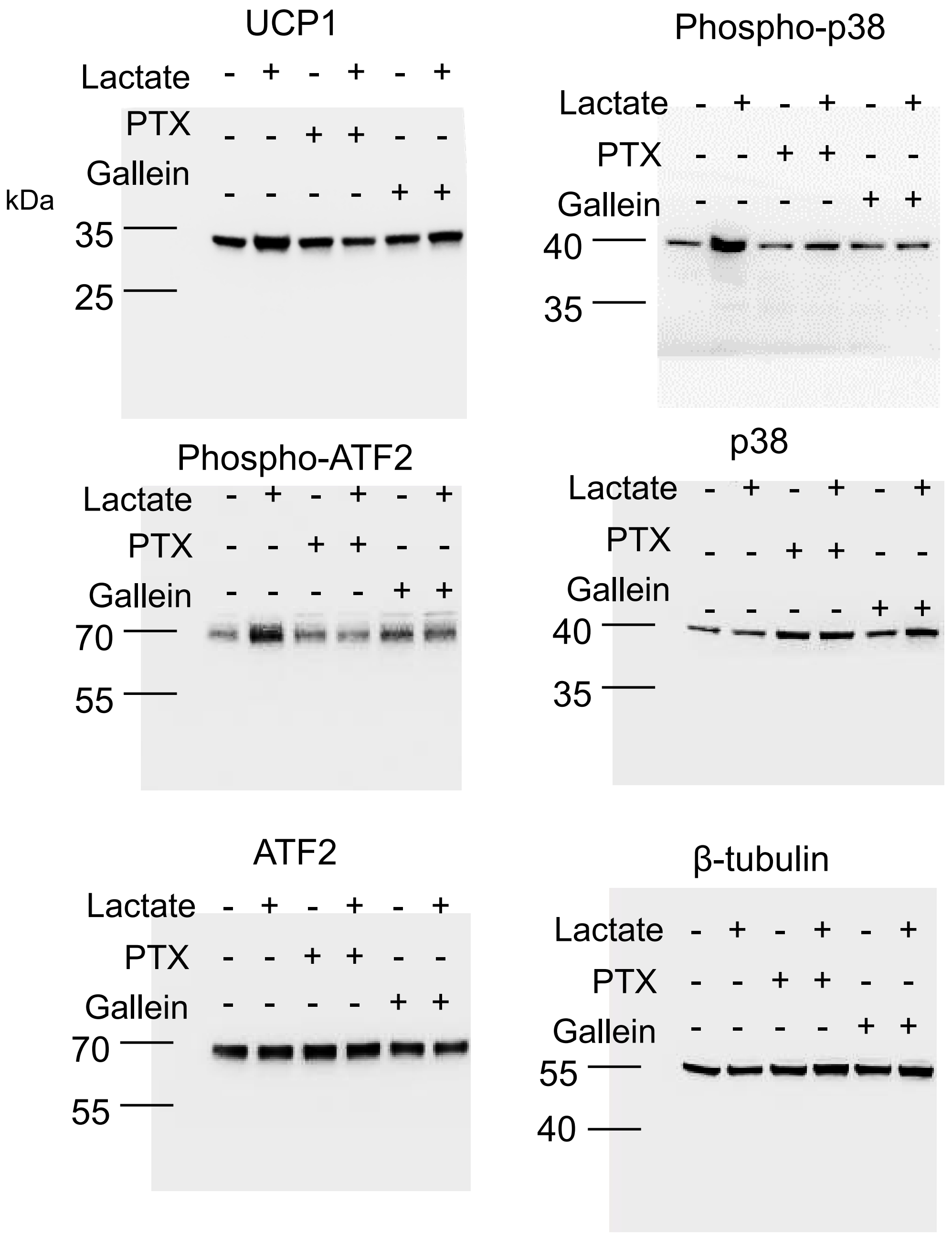

Fig. 6c: UCP1, phospho-ATF2, ATF2, phospho-p38, p38 and β-tubulin.

Raw data of western blots in Fig. 6d

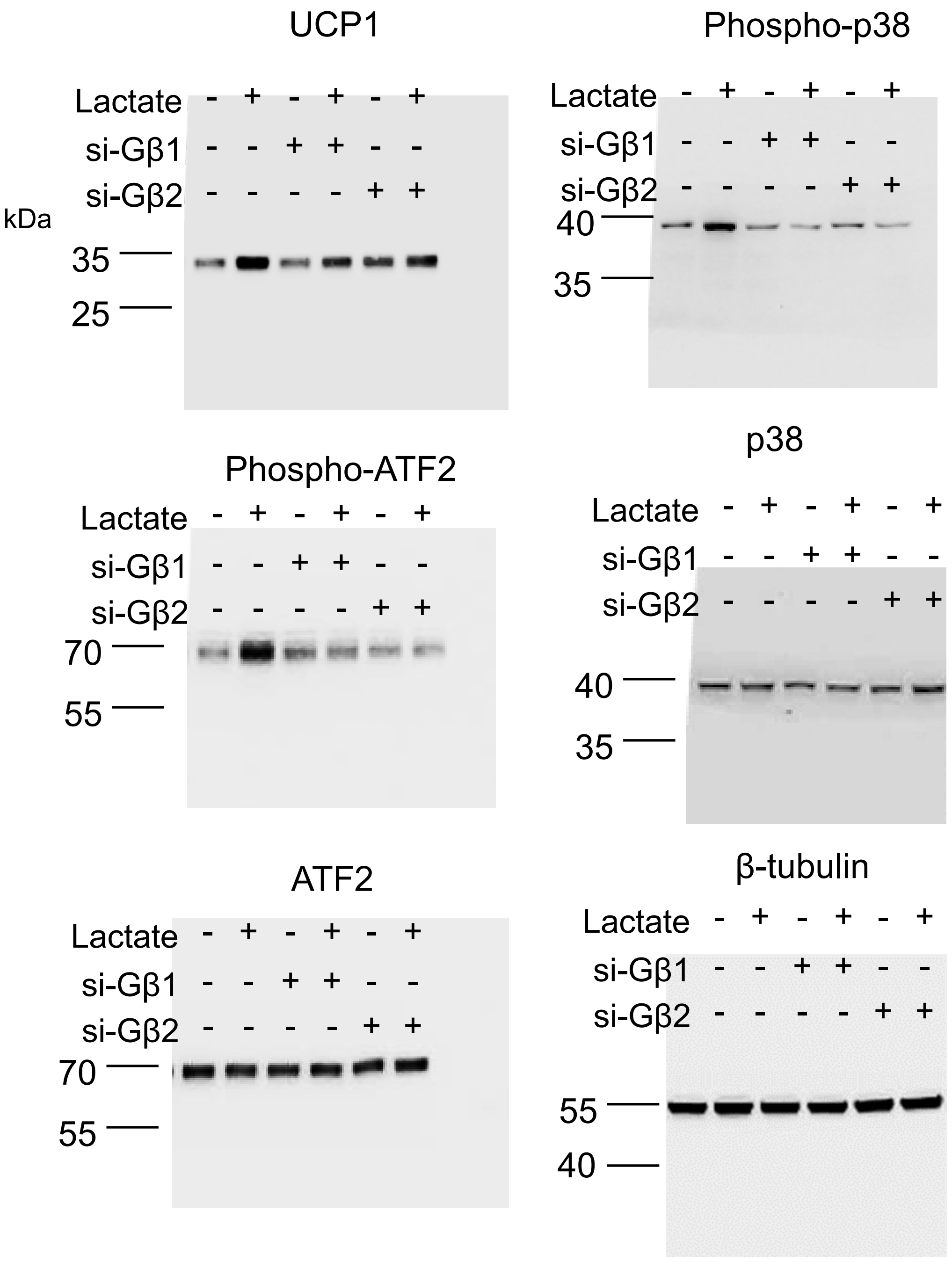

Fig. 6d: UCP1, phospho-ATF2, ATF2, phospho-p38, p38 and β-tubulin.

Raw data of western blots in Fig. 6f

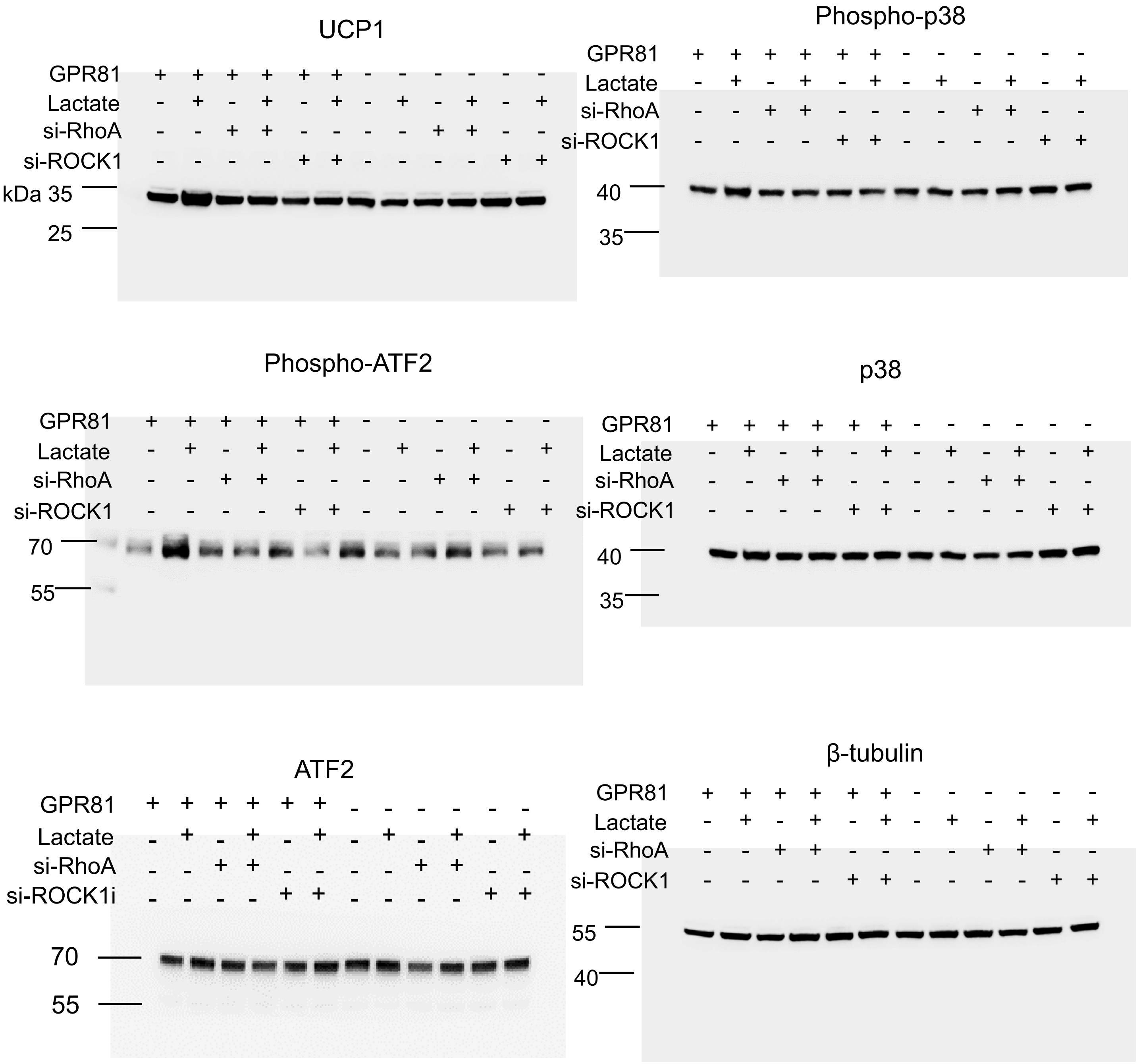

Fig. 6f: UCP1, phospho-ATF2, ATF2, phospho-p38, p38 and β-tubulin.

Raw data of western blots in Fig. 6g

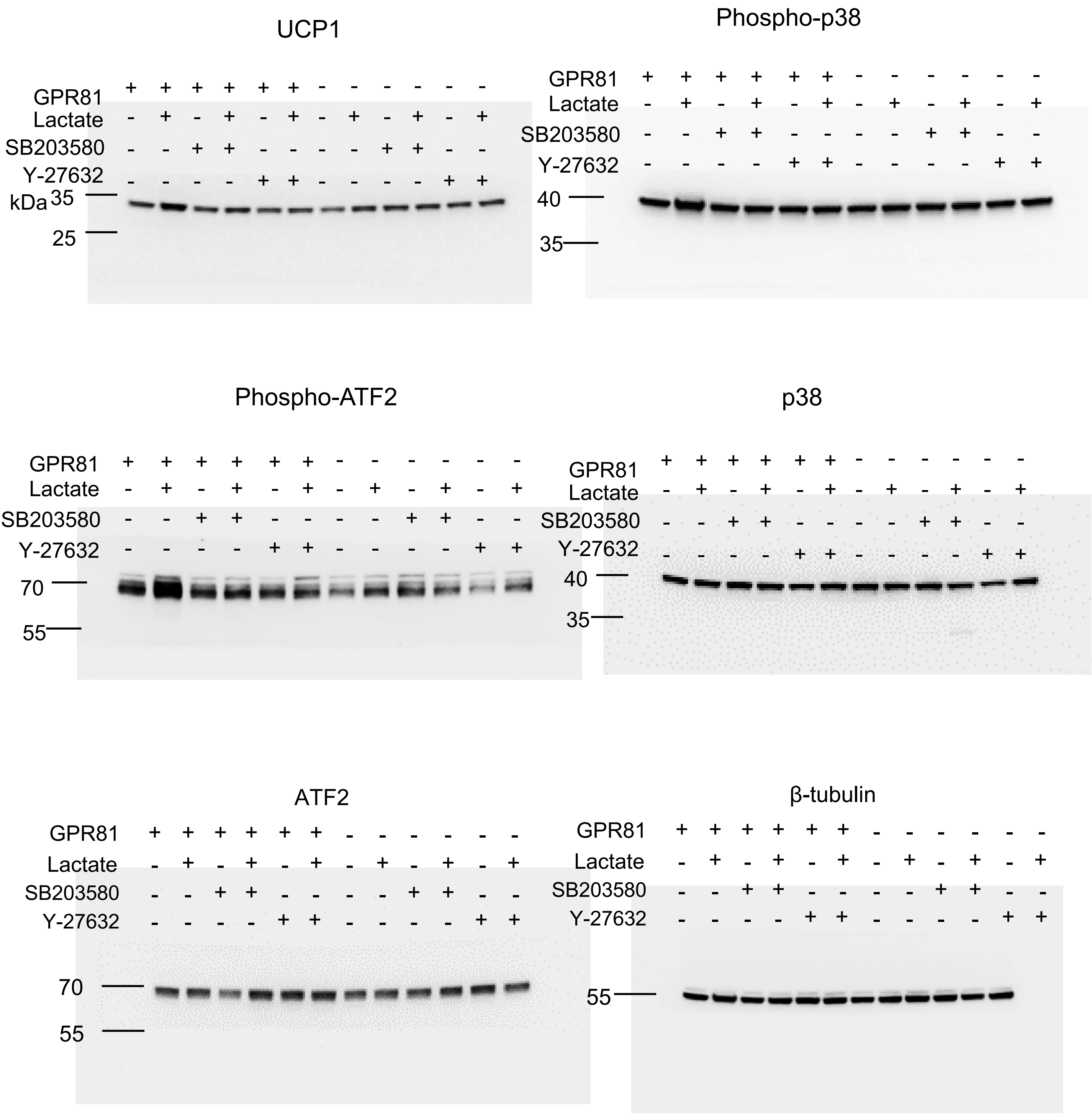

Fig. 6g: UCP1, phospho-ATF2, ATF2, phospho-p38, p38 and β-tubulin.
